# Neuropsychological and metabolic interconnectivity in obesity, anorexia and bulimia nervosa – an integrative literature review

**DOI:** 10.1007/s11011-026-01892-y

**Published:** 2026-07-08

**Authors:** Malini Turner, Mansi Dass Singh, Ian Evans

**Affiliations:** University of New England, Armidale, NSW 2350 Australia

**Keywords:** Neurobiology, Metabolism, Bi-directional signalling, Brain-gut axis, Brain-gut-adipose axis, Brainstem, Hypothalamus, Neuropeptides, Obesity, Anorexia nervosa, Bulimia nervosa, Mental health

## Abstract

A dysfunctional bi-directional signalling of plural neural networks expresses distinct metabolic disruption with mental health consequences in obesity, anorexia nervosa and bulimia nervosa. Maladaptive brain-gut connectivities lead to multifactorial contributing factors raising the interest of researchers in an effort to address their neurobiological, psychological and metabolic factors to improved mental health outcomes. The first aim of this review was to collate clinical evidence on brainstem-hypothalamus pathways in obesity, anorexia nervosa and bulimia nervosa. Further, it sought to describe the chief brain-based interactions within both the brain-gut and brain-gut-adipose axis in these conditions. Another aim was to explore the interactions of prominent peptides within the brain-gut and brain-gut-adipose axes. The final aim was to integrate the knowledge of maladaptive neural, peptide and hormonal signalling interactions with the mental faculty. According to integrative review guidelines, the multileveled information was grouped into three superordinate themes: the brain neurofeedback, the stomach neurofeedback and the sympathoadrenal neurofeedback, with seven subordinate themes: brain stem, lateral nucleus of the hypothalamus, arcuate nucleus of the hypothalamus, mechanism of appetite regulation, short-term satiety and long-term satiety signalling as well as the mechanisms of glucoprivation and lipoprivation, presented in Table 1. Their interconnectivites are synthesised in seven Figures, presented at each subtheme section. This paper augmented our understanding of brain maladaptive interactions with gut peptides and hormones among people with obesity and eating disorders and may serve a roadmap to neurobiological and metabolic influences on physical and mental health. Limitations identify qualitative areas of research towards evidence-informed psychiatric and health counselling support.

## Introduction

The neural connectivity between the brain and body weight through food intake, energy expenditure, body fat stores, and endogenous glucose production has been of interest to the health professionals for a long time. Obesity, anorexia nervosa and bulimia nervosa are complex conditions with low rates of detection and low treatment uptake, requiring an understanding of their neurometabolic and mental health factors to enable early recognition of patterns and lead to early interventions, essential in prevention of further psychopathology and a reduction of relapse (Austin et al. 2021; Fatt et al. 2020; Koreshe et al. 2023).

Epidemiological studies suggest that obesity factors are on increase, with comorbidities and increased mortality sequelae (Hruby & Hu 2015). While genetic predisposition to abnormal metabolism is one well-known contributing factor, stress-induced, uncontrolled food intake is an emerging major contributor to weight gain with some individuals inherently more susceptible to the negative effects of stress than others (Diz Chaves 2011; Tomiyama 2019; van der Valk et al. 2018). Because the brain-gut neural axis is involved in the disruption of goal-directed behaviors (Yamada et al. 2018), the growing understanding of the brain-based influences on eating behaviours indicates a derangement across various layers of brain-gut axis and a coherent presentation of these mechanisms could contribute to more precise choices to treatment. A correct understanding and application to this complex condition of abnormal emotional functioning, and depressive symptomatology is important, because research is clear that obese people avoid seeking treatment largely due to wide-spread stigmatisation and associated shame (Chakravorty 2021; Dallman, 2010).

In Australia, there has been a six-fold increase in prevalence of eating disorders since the late 1990 s (National Eating Disorders Collaboration 2023). Eating disorders adversely affect a person’s physical and mental health with neuroscience research indicating altered brain structure and function as a common features of both anorexia nervosa and bulimia nervosa (McAdams and Smith 2015). Anorexia nervosa is characterised by a low body mass index (BMI), starvation due to fear of gaining weight, denial of current low weight and by serious negative impact on mental health (American Psychiatric Association [APA] 2013), with amenorrhea occurring in the majority of female patients (Mustelin et al. 2016). Despite the evidence of neurophysiological malfunctions, anorexia -afflicted individuals do not feel any change in themselves and do not appear distressed, further lacking acknowledgement of their condition during consults and refusing treatment for underweight (Casper 2022). Physiologically, bulimia nervosa’s characteristic symptoms include large amounts of food intake (binge-eating behaviour) after prolonged fasting, following episodes of self-induced vomiting (purge behaviour) (Albracht-Schulte et al. 2023) or excessive use of laxatives (APA 2013). However, bulimia nervosa patients eat normally with friends and family and only binge-eat when alone with more severe cases, altering their daily routines to make time for the binge and purge outlets (Rushing et al. 2003).

Diagnostic and Statistical Manual of Mental Disorders, Fifth Edition (DSM-5) suggests medical treatment interventions in late discovery or advanced cases of eating disorders (APA, 2013). Theoretically, serotonin fluctuations are known to disturb appetite, simultaneously leading to anxious and obsessive behaviours, both chiefly based on lack of impulse control (Cloninger 1987). Brain imaging research in both these conditions has repeatedly implicated activation of the brain structures related to meal-sizes, taste-reward and salience-processing regions (Berthoud et al. 2006; Donnely et al. 2018; Frank et al. 2016; South and Ritter 1983). However, starvation is known to increase stress by activating the HPA axis (Schmalbach et al. 2020) and the symptoms of anorexia nervosa are based on increased HPA axis functions, displaying elevated hunger-signalling neuropeptides (Comeras et al. 2019; Pannicke et al. 2021), simultaneously demonstrating fear and avoidance behaviours (Steinglass et al. 2011). The neuroscientific view of such outcomes is based on feedback from the brain, stomach and sympathoadrenal system hunger-satiety malfunctional pathways, affecting adequate production of brain-gut-adipose axis hormones and peptides, resulting in pathogenesis of eating disorders (Smitka et al. 2013).

## Methodology

### Methods and data evaluation

A narrative integrative literature review structure is based on five stages, namely problem identification, literature search, data evaluation, data analysis and presentation of findings, fundamental to the rigor of the integrative studies (Ferrari 2015; Sukhera 2022; Whittemore and Knafl 2005). The main search was conducted with a primary focus on clinical studies, systematic reviews, meta-analyses and other integrative reviews, seeking to meet the four aims of this study such as gathering clinical evidence to inform all the to-date neural, metabolic and mental health factors, known to be prevalent across studies on both obesity and eating disorders. While seeking to present brain-based mechanisms for food intake, the peptide and hormonal interactions within the brain-gut and brain-gut-adipose axes, the review further at creating a platform for evidence-informed, person-centered treatment. Such platform was considered to best suit the focus on pragmatic understanding and the empirical application of outcomes (Jahan et al. 2016). This provided a platform for the next aim which was achieving thoroughness in an effort to create an overarching holistic perspective (Hopia et al. 2016), recommended in both psychiatric and health counselling work. As the bi-directional effects of the neurometabolic as well as mental health changes involve a complex understanding of both the client and the formation of clinical approach, the final aim of this work centred only on the prominent tenets for practicality and feasibility, particularly for the novice psychiatric and counselling practitioners. As such, this narrative integration does not claim to be exhaustive however, focus remained on selected medical and biological literature that supported clear statements aiming to make way for qualitative inquiries in the field (Chigbu 2019).

A narrative integrative literature review accommodates for insights into the current progress of a given field and typically, does not adhere to inclusion and exclusion criteria (Sukhera 2022). For the purpose of practicality, this study kept clear boundaries between thinking and interpretation (Greenhalgh et al. 2018) which however, were specific enough to allow for a subjective direction during data evaluation (Sukhera 2022) such as asking the question: “Could the psychiatric or health counselling professional use the outcomes of this work in their practice?” Adhering to this structure, another aim of this study was to capitalise on critical thinking and interpretation of *evidence-informed* rather than *evidence-based* work (Nevo and Slonim-Nevo 2011). This factor alone supported the rationale behind the structure of this review as evidence-informed health outcomes are conducive to the novice clinicians, critical thinking in their decision-making and referral processes.

### Literature search

This literature review began in March 2022 and was completed in October 2025, using the Boolean phrasing with the key words: (eating disorder OR obesity OR anorexia OR bulimia) AND (neurobiology) AND (brain OR “brain metabolism” OR “brain area’ OR gastro* OR “brain gut” OR “brain-gut-adipose”) AND (hunger OR “hunger mechanism” OR satiety) AND (hormon* OR neuro* OR peptide). The key search terms were applied across PubMed and Google Scholar and included journal articles published in English between 1990 and 2025. The complex information was grouped into three superordinate themes and seven subthemes, presented in Table 1.

**Table 1:** Themes to neurometabolic factors in obesity, anorexia nervosa and bulimia nervosa

Superordinate Themes	Subthemes
1. Brain neurofeedback	1.1. The brain stem 1.1.1. Dorsal vagal complex 1.1.1.1. Nucleus of solitary tract 1.1.1.2. Area postrema 1.1.1.3. Vagal dorsal motor nucleus (see Figure 1). 1.2. The hypothalamus 1.2.1. Lateral nucleus of hypothalamus 1.2.1.1. α-MSH, MCH, AgP and PYY signals in starting a meal (see Figure 2). 1.2.2. Arcuate nucleus of hypothalamus 1.2.2.1. NPY, POMC, CART and MCH signals in regulation and completion of a meal (see Figure 3).
2. Stomach neurofeedback	2.1. Starting of a meal: Ghrelin, NPY, CART and AgP signals in appetite regulation (see Figure 4). 2.2. Completion of a meal: Cholecystokinin and PYY signals in short-term satiety from stomach, duodenum and liver (see Figure 5). 2.3. Fasting: Leptin signalling in long-term satiety from adipose tissue (see Figure 6).
3. Sympathoadrenal system	3.1. Chronic changes in glucose levels and fatty acid oxidation: the mechanisms of glucoprivation and lipoprivation (see Figure 7).

**Box 1: **Table 1

## Neurometabolic connectivities in in obesity, anorexia and bulimia nervosa

### Brain neurofeedback

#### The role of the brainstem 

Brain stem contains neurons that detect hunger and satiety signals and produce behaviours of acceptance or rejection of food. Connections from brain stem to forebrain directly influence metabolic rate and impulse for or rejection of, food intake (Miller 2019). Nucleus of solitary tract, area postrema and vagal dorsal motor nucleus are the three chief parts of brain stem that connect peripheral nervous system with hypothalamus (Da Silva and Bloom 2012) and they are referred to as *dorsal vagal complex* (Cheng et al., 2022; Mirza and Das 2019).

#### Nucleus of solitary tract

**Fig. 1:**
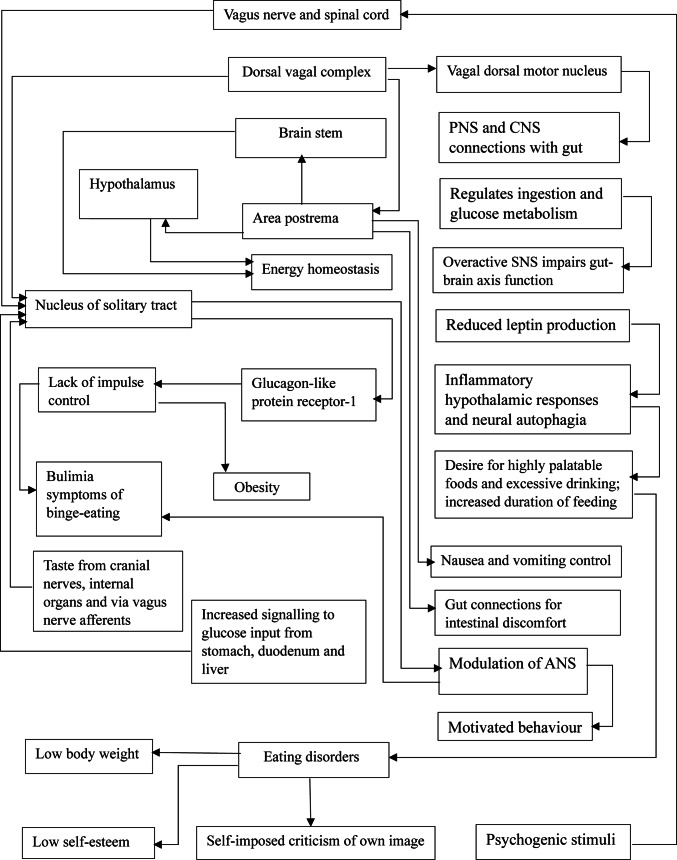
Brain neurofeedback via brainstem dorsal vagal complex: Nucleus of solitary tract, area postrema and vagal dorsal motor nucleus. ANS: autonomic nervous system; PNS: peripheral nervous system; SNS: sympathetic nervous system

Nucleus of solitary tract receives taste input from the cranial nerves and receives afferent signals from various organs via vagus connections (Mirza and Das, 2019). It was found to exhibit particular affinity to glucose modification by demonstrating firing rates corresponding to varied glucose intake levels from stomach, duodenum, and liver (Roberts et al. 2017). Because nucleus of solitary tract receives afferent impulses not only from vagus nerve but from the spinal cord as well, it has a capacity to be activated by a number of psychogenic stimuli (Holt 2022). As a result, it modulates the autonomic nervous system output towards a motivated behaviour (Maniscalco and Rinaman 2018). Motivated behaviour is an important point in obesity and eating disorders, because of its explicit focus on self-imposed critical attitudes to image accompanied with low self-esteem (Casper 2022), as seen in Fig.1

#### Area postrema

Area postrema is adjacent to the nucleus of solitary tract and plays a crucial part in hunger by facilitating neuronal connections between the gut hormones, brainstem and forebrain circuitry (Ahima and Anthwi 2008). Its neuronal communication is largely facilitated by the absence of a full blood–brain barrier which increases molecular permeability (Da Silva and Bloom 2012). The fenestrated capillaries of hypothalamic median eminence allow hormonal and nutrient access to both the brainstem and hypothalamus, contributing to energy homeostasis (Miller 2019; Peruzzo et al. 2000). Area postrema plays a prominent role in controlling nausea and vomiting, chiefly because of its dense population with glucagon-like protein receptor-1 from the gut, processing sensory information and invoking intestinal discomfort (Kawatani et al. 2018; Trapp and Brierley 2022). Glucagon-like protein receptor-1 is of emerging research interest due to its large neural population in the nucleus of solitary tract, with current studies linking its aberrant signalling in that area, to low impulse control, resulting to pathways of binge eating conditions such as obesity and bulimia nervosa (Alhadeff et al. 2017), as shown in Fig. 1.

#### Vagal dorsal motor nucleus

The vagal dorsal motor nucleus regulates ingestion and glucose metabolism (Holt 2022). Its activity is mainly parasympathetic and as such, it stimulates the function of the organs of the abdominal cavity (Zsombok et al. 2014). Vagus nerve receptors densely populate gastrointestinal area and link the peripheral nervous system with the central nervous system (Browning et al., 2017; Greene, 2014). Since vagal dorsal motor nucleus is downregulated via overactive sympathetic activity (Breit et al. 2018), it reduces the adequate function of leptin receptors. Such disrupted exchange downregulates the gut-brain axis signalling and impairs leptin transportation across the blood–brain barrier, causing hypothalamic inflammation along with neuronal autophagic activity (Meng and Cai, 2011; Myers et al., 2012). Such maladaptive signalling results in overconsumption of highly palatable food and excessive drinking, accompanied by a consistent low body weight (South and Ritter 1983) as well as an increased duration of feeding time (de Silva and Bloom 2012), as shown in Fig 1.

**Box 2: **Fig. 1

#### The role of the hypothalamus

The hypothalamus moderates homeostasis by connecting central nervous system (CNS) to the enteroendocrine and sympathoadrenal systems, and modulates heart rate, appetite, thirst, temperature, and the release of gut peptides ghrelin and leptin as well as the hormone insulin (Thau et al. 2022). Two hypothalamic areas play role in hunger and satiety: the lateral nucleus of hypothalamus, connected to acceleration of hunger and the arcuate nucleus of hypothalamus related to both appetite stimulation and inhibition (Roger et al. 2022).

#### Lateral nucleus of the hypothalamus: α-MSH, MCH, AgP and PYY signals in starting a meal

**Fig. 2:**
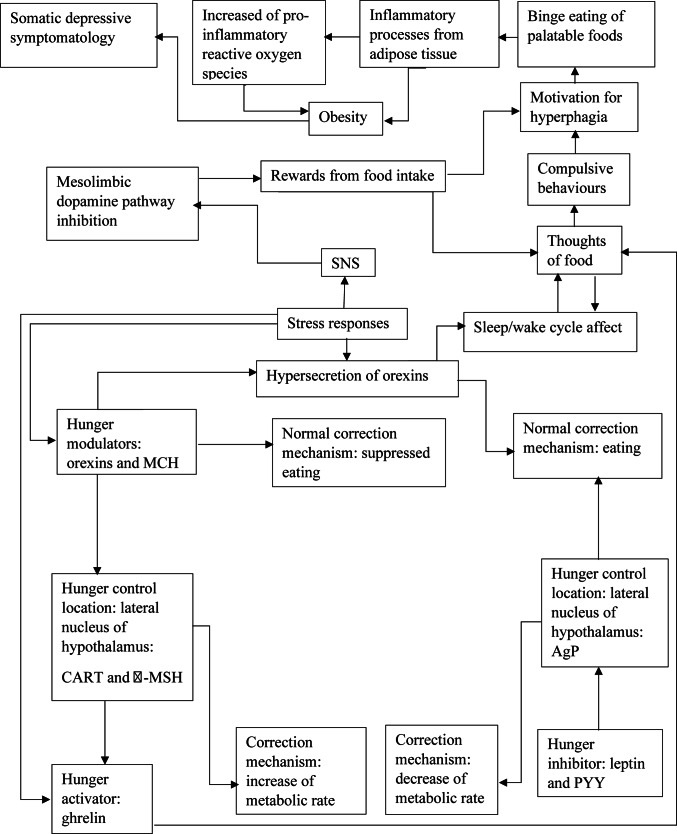
Lateral nuclei of hypothalamus: α-MSH, MCH, AgP and PYY signals in starting a meal α-MSH: alpha-melanocyte-stimulating hormone; AgP: agouti-related protein; CART: cocaine- and amphetamine-regulated transcript; MCH: melanin-concentrating hormone; PYY: peptide tyrosine tyrosine; SNS: sympathetic nervous system

The circuitry of the lateral nucleus of the hypothalamus chiefly consists of orexin and melanin-concentrating hormone (MCH) neural populations (Dilsiz et al. 2020). Orexins are of neuromodulating activities (Nixon et al. 2012) and are regulated by neurotransmitter systems and environmental stimuli, both related to reward-seeking behaviours, including food intake (Mohammadkhani et al. 2024). MCH is a neurological complex that maintains energy intake and expenditure to promote energetic balance after caloric consumption (Lord et al. 2021). Together with orexin, MCH maintains a pivoting role in engagement and regulation of food intake (Hahn 2010). As shown in Fig. 2, the processes of food intake and appetite suppression are bi-directional because the peripheral peptides and hormones influence sympathetic nervous system (SNS) that in turn, activates the metabolic phases (Austin and Marks 2009). For example, as both sensory and motor activator of the brain-gut neural axis, the bi-directional signalling of the vagus nerve plays a role in moderation of energy intake and expenditure (Yahagi 2017). Further, as orexigenic pathways play a vital role in energy balance, their interaction with the gut peptide ghrelin is focused on moderation of energy expenditure (Ferno et al. 2015). Because ghrelin signalling occurs simultaneously with the neuropeptide signals for lowering fat metabolism, the ghrelin effects, transmitted via vagal nerve, are to trigger thoughts of food.

Hypersecretion of orexins translates into two systems affect: disturbances in sleep–wake cycles (Hara et al. 2005; Kotz et al. 2012), and deficiencies in energy balance (Dahmen et al. 2008), (see Fig. 2). This could be due to the conflictual signaling between two dysfunctional pathways - one of an acute and one of a chronic depletion of orexin signalling. For example, higher levels of serum orexin interfere with spontaneous physical activity and the non-exercise thermogenesis, contributing to the development of oxidative stress, common in ischaemic conditions which are considered the underlying mechanism to obesity resistance transcription factors (Butterick et al. 2013). Increased concentrations of MCH lead to hyperphagic behaviours as the acute phase of MCH neuron firing increases food intake (Noble et al. 2019) while MCH chronic signalling leads to expansions in body weight, overtime (Mul et al. 2010). (see Fig. 2).

MCH participates in stress responses as well as in mood modulation (Al-Massadi et al. 2021) and is involved in reduction of appetite and increased energy expenditure via its connectivity with the melanocortin receptor 4 (Tao 2010). As MCH has simultaneous connectivity with the dopamine receptor 2 (Concetti et al. 2023), it acts as a specific dopamine depressant (Conductier et al. 2011). Given these dual signalling, MCH neuronal activation is responsible for feelings of rewards from food and for the reinforcement of ongoing food intake (Dilsiz et al. 2020) with its aberrant signalling having selective effects on the mesolimbic dopamine pathways (Pissios et al. 2008). Since both melanocortin receptor 4 and dopamine receptor 2 interact to respond to food rewards (Yoon and Baik 2015) and both densely populate the mesolimbic dopamine pathway, their abnormal connectivity reinforces compulsive behaviours, generating motivation for food reward via binge eating accompanied with choices of highly palatable foods (di Bonaventura et al. 2020). Due to MCH prominent bi-directional signalling in obesity and eating disorders, low thermogenesis, oxidative stress and ischaemic conditions suggest possible connectivity to somatic depressive symptomatology on account of pro-inflammatory cytokine signalling from the adipose tissue, increasing production of reactive oxygen species (Marseglia et al. 2014; Turner 2024). Figure 2 shows how in such connectivity, the neuronal circuits in reward-based feeding increase motivation for palatable foods intake. The resultant pleasure from this process leads to surrendering to the impulse for food intake thereby prioritising sleep deprivation in favour of eating (Soltanieh et al. 2021).

**Box 3: **Fig. 2

#### Arcuate nucleus of the hypothalamus: NPY, POMC, CART and MCH signals in regulation and completion of a meal

**Fig. 3:**
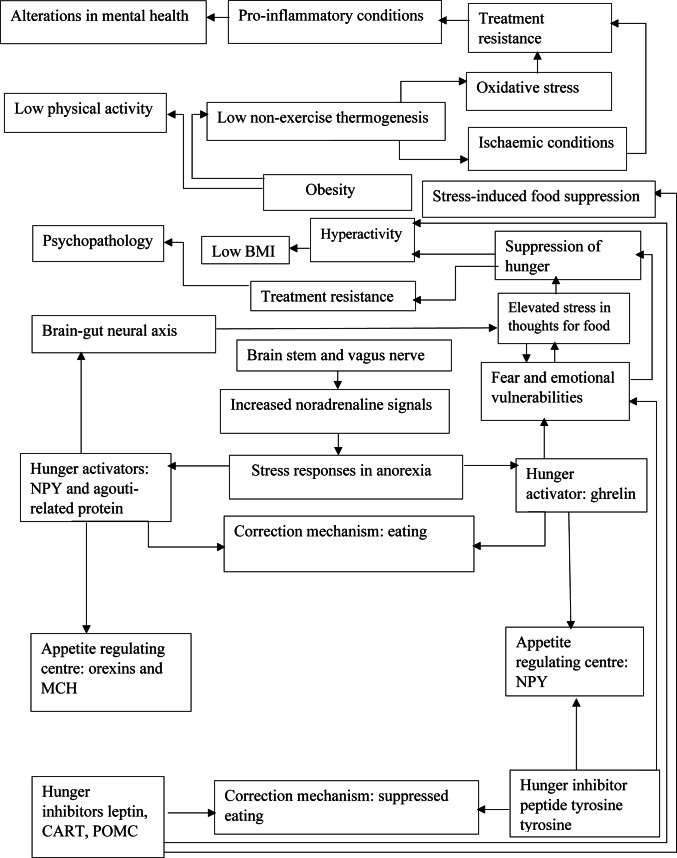
Arcuate nucleus of hypothalamus: NPY: neuropeptide Y; CART: cocaine- and amphetamine-regulated transcript; POMC – pro-opiomelanocortin; MCH: melanin-concentrating hormone; BMI – body mass index

The arcuate nucleus of hypothalamus has strategic systems that regulate appetite by expressing several neuronal groups. Neuropeptide Y (NPY) and agouti-related proteins (AgP) act as neuromodulators (Hirsch and Zukowska 2012) as well as behavioural modulators (Ferno et al. 2015; Tanaka et al. 2021) as shown in Fig. 3. NPY is a major hunger activator, linked to the brain nutrient status (Comeras et al. 2019). In normal conditions, the role of AgP is to detect nutrient requirements, overcome food-suppressive signalling, decrease activity of anorexigenic neurons and initiate food-seeking behaviour (Essner et al. 2017). AgP is a neuropeptide that holds a dual role: firstly, it interacts with NPY to increase eating and decrease body metabolic rate and secondly, it acts on melanocortin receptors 4 to maintain appetite and thermogenesis by lowering energy expenditure (Ilnytska and Argyropoulos 2008). NPY and AgP both play a pivotal role in energy balance and maintenance of body weight (Jackson et al. 2006; Vohra et al. 2022) (see Fig. 3).

The neuropeptides pro-opiomelanocortin (POMC) and cocaine- and amphetamine-regulated transcript (CART) inhibit feeding (Murphy and Bloom 2006; Neudorfer et al. 2020). CART exhibits appetite suppression effect becasue its receptors are chiefly located in arcuate nucleus of hypothalamus, where its terminal connections send and receive satiety responses (Thim et al. 1998). CART holds a neuronal affinity to central and peripheral glucose-sensing sites, and fires for selective food intake to an increased energy expenditure (Lau and Herzog 2014). The CART network chiefly involves the melanotropin (α-melanocyte-stimulating hormone), which is an anorexigenic (appetite-suppressing) complex (Tian et al. 2004). This hormone is related to adrenocortical hormonal secretions and in particular, to the production of the glucocorticoid hormone cortisol (Anderson et al. 2016). CART inhibits food intake and modulates both insulin secretion and lipid metabolism (Morgan and Cone 2006). As AgP, NPY and CART are sensitive to cortisol (Schwartz et al. 2017), they exchange signals across the brain-gut neural axis, influencing the brainstem and vagus nerve (Grill and Hayes 2012), thus affecting cognitive complexes such as values of food, food preferences as well as invoking food-related memories (Kanoski et al. 2011). Studies suggest that anorexia nervosa exhibits a continuously elevated levels of NPY, indicating a permanent status of a search for food as well as ability to consume large amounts of it (Södersten et al. 2008; Tyszkiewicz-Nwafor et al. 2021). This mechanism keeps noradrenergic signalling from the brain stem to the arcuate nucleus of hypothalamus at a constant operation (Nagatani et al. 1996), given that CART is well-known contributor linked to anorexia nervosa hyperactivity factor (Jean et al. 2012). POMC has recently been confirmed to contribute to stress-induced hypophagia resulting in overall anhedonia (Qu et al. 2020). Because CART and POMC signals involve both the motor and sensory neuronal activities, the body stress levels are kept permanently high (Lach et al. 2018). As ghrelin plasma levels have been positively associated with physical exercise, hyperactivity is likely to be a strong predictor of elevated ghrelin levels (Schalla and Stengel 2018). The brain-gut neural axis connectivity translates this process in mental symptomatology such as fear-based emotional vulnerabilities leading to self-inflicted food restrictions and resulting in low body mass index (Strober 2004). Hyperactivity and depressive moods resulting from voluntary food suppressions increase the risks of stress-exacerbated neural, physiological and psychopathologies in eating disorders as shown in Fig. 3.

**Box 4: **Fig. 3

## Stomach neurofeedback

### Starting of a meal: Ghrelin, NPY, CART and AgP signals in appetite regulation

**Fig. 4:**
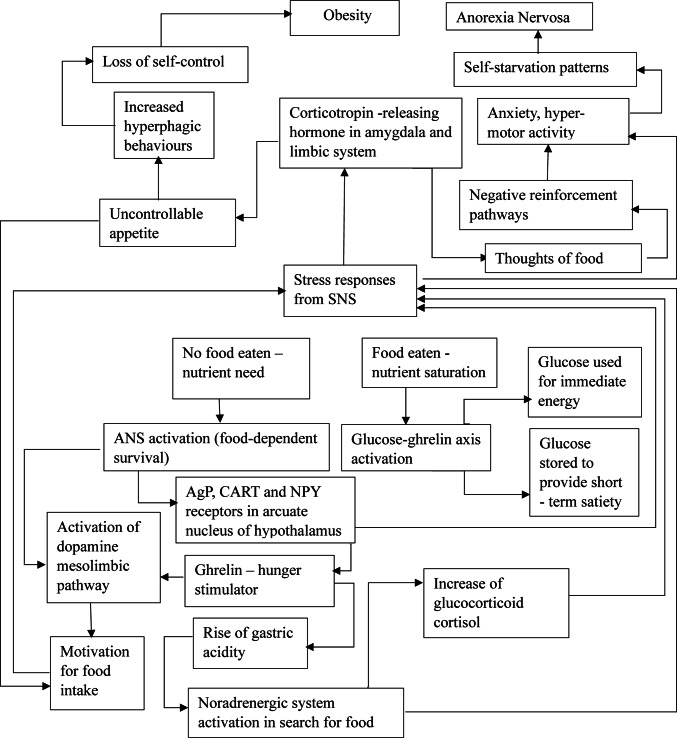
Starting of meal: Ghrelin, NPY, CART and AgP signals in appetite regulations AgP: agouti-related protein; CART: cocaine- and amphetamine-regulated transcript; NPY neuropeptide Y

Two major signals are of physiological significance in controlling food intake: the first is whether the stomach is empty, determining return of hunger and the second is how much food has been already eaten (Tack et al. 2021). When the stomach is empty, it releases peptide ghrelin which is an orexigenic hormone and a short-term hunger stimulus peptide that is physiologically related to production of growth hormone (Wang et al. 2008). Blood plasma ghrelin increases before meal intake times and it is generally activated with food entrance in the duodenum along with rise of the acidic gastric secretion (Diz Chavez 2011). Ghrelin is predominantly suppressed via glucose-ghrelin axis neurotransmission during short-term metabolic phase when the amount of food consumed has been determined sufficient for the body’s needs (Mani et al. 2019). Some of the glucose is utilised and some of it is stored in the liver in form of short-term energy reservoir, until the next food intake (Chun-Xia and Tschop 2012) (see Fig. 4).

In obesity and eating disorders, brain-gut neurocircuitry chiefly involves dopamine cells of ventral tegmental area and their projection to nucleus accumbens, both strongly activating food-rewarding behaviours (di Bonaventura et al. 2020), simultaneously increasing ghrelin release for hunger thusenhancing motivation for food (Smitka et al. 2013). As short-term feeding phase is observed to enhance the production of cortisol and insulin, it simultaneously reduces aberrant brain activity related to stress-responses (Kumar et al. 2022; Tyszkiewicz-Nwafor et al. 2021). This behavioural mechanism is of a particular interest in obesity research, as ghrelin activation in dopamine mesolimbic pathway directly stimulates motivation and food reward circuits and results in hyperphagic behaviour with a contribution to adipose tissue growth (Hanssen et al. 2022). Hyperphagic behaviours further increase the concentration of the glucose-dependent cortisol in the blood, that may result in uncontrollable, stress-based, obesity-enhancing behaviours (Dallman 2010). Simultaneously, raise of blood serum glucocorticoids stimulates corticotropin-releasing hormone in the amygdala and other limbic sites, impacting the monoaminergic neurons in the brainstem to initiate motivated behaviours (Dallman 2010; Ulrich-Lai and Herman 2009). Further, hunger is primarily perceived by AgP in the arcuate nucleus of hypothalamus, as ghrelin receptors on AgP neurons increase plasma concentration of ghrelin (Méquinion et al. 2013). This mechanism activates brain-gut pathway as both peptides and neurotransmitters increase eating (Diz-Chavez 2011)(see Fig. 4). 

AgP, NPY and CART are sensitive to ghrelin release (Morrison et al. 2005). As AgP is known to facilitate a range of behaviours such as motivation, locomotion, anxiety and overall negative reinforcement, people with anorexia nervosa show increased activity across ghrelin signalling pathways, resulting in thoughts of food (Jerlhag et al. 2006). However, the prolonged food restriction symptom of anorexia nervosa leads to a possible long-term ghrelin insensitivity (Schalla and Stengel 2018), resulting in a dysfunctional signalling with metabolic consequences such as a negative energy balance on the brain-gut axis (Grill and Hayes 2012) (see Fig. 4). This neurobiological observation was linked to mental health deviations such as hyperactivity and addiction to self-starvation (Méquinion et al. 2013).

**Box 5: **Fig. 4

### Completion of a meal: Cholecystokinin and PYY signals in short-term satiety from stomach, duodenum and liver

**Fig. 5:**
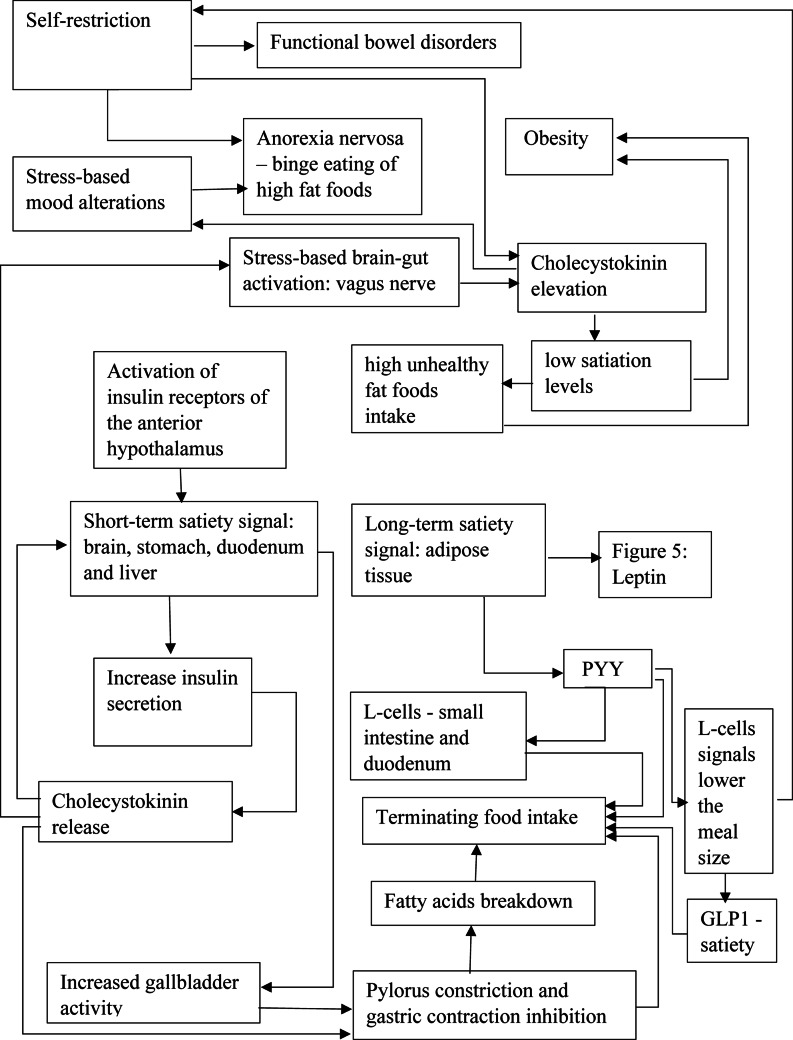
Completion of a meal: Cholecystokinin and PYY signals in short-term satiety. GLP-1: glucagon-like peptide 1; PYY: peptide tyrosine tyrosine

Two types of satiety signalling stop a meal: the first is short-term satiety signal, involving the brain, stomach, duodenum and liver and the second is long-term satiety signal, involving the adipose tissue (Barakat et al. 2024). During short-term satiety, insulin secretion is increased, allowing the body to absorb nutrients other than glucose. Certain amount of insulin is required to travel through blood–brain barrier in order to trigger action in the insulin receptors. Located in the anterior hypothalamic region, their neuronal population detects the rising levels of insulin in the body, and releases signal, inhibiting hunger (Blasquez et al. 2014). During food ingestion, the stomach nutrient receptors to satiety detect the levels of food taken, and signal termination of intake when the body needs have been met. Once the food is mixed with bile and pancreatic enzymes, cholecystokinin is released. Metabolically, cholecystokinin is known as hormone that breaks down the fatty acids by triggering simultaneous pylorus constriction and gastric contraction inhibition (Raybould 2007). Since cholecystokinin has neuronal receptors that inhibit hunger by signalling meal satiety, its secretion increases gallbladder activity, simultaneously preventing duodenum from giving more food, thus terminating eating behaviours (Okonkwo et al. 2020). The action of cholecystokinin across the brain -gut axis remains primarily dependent on the vagus nerve, assuming to lead to decreased duration of food intake, smaller sizes and functional cues to induction of satiety (Peters et al. 2006). Cholecystokinin interactions are shown in Fig. 5.

Various clinical research during 90’s indicated that cholecystokinin is elevated during eating disorders (Geracioti et al 1992; Fujimoto et al. 1997; Phillipp et al. 1991). For example, metabolically high levels of cholesystokinin were linked to increased levels of cholesterol and physiology-based abnormalities (Powell-Wiley et al. 2021). While on a neural level, the vagus nerve afferent connectivity signals low satiation levels and continued intrinsic enforcement for high saturated and trans-fat foods intake to energetic balance (de Lartigue et al. 2011), with both factors leading to obesity. In the gastrointestinal tract, cholecystokinin acts as a major peptide and its elevated levels have been implicated in a number of gastrointestinal pathologies. such as reduced ability to downregulate SNS to relaxation with CNS disturbances, thus increasing stress-based mood alterations in anorexia psychopathology (Breit et al. 2018; Greene 2014; Schorr and Miller 2017; Sjolund et al. 1996).

Another example is self-restriction behaviours, effecting increase of serum cholecystokinin simultaneously reducing satiation rates (Bailer and Kaye 2003). Because of the interconnectivity of arcuate nucleus of hypothalamus via brain-gut axis (see Fig. 3), anorexia nervosa individuals are vulnerable to binge eating episodes with a prominent loss of self-control when presented with food (Cuntz et al. 2013). Along with the impredictability of altered mood levels, the cholecyctokinin mechanism malfunction plays a role in anorexia nervosa irregular and idiopathic bowel movements and functional bowel disorders as well as pathogenesis of inflammatory bowel disease (Larsen et al. 2021; Tang et al., 1998) as seen in Fig. 5.

The anorexigenic peptide, PYY is normally low after a prolonged starvation, indicating a need for food intake (de Silva and Bloom 2012). It is found in adipose tissue as well as in the small intestine and it is the chief activator of L-cells located between stomach and duodenum (Lach et al. 2018). Since L-cells’ only response is to decrease the size of meals ingested, their signalling inhibits the feeding process, leading to decreased bodily metabolism (Vincent and le Roux 2008). Because L-cells produce glucagon-like peptide (GLP-1) that signals satiety, this mechanism of action terminates food intake, making it essential for glucose homeostasis, pivotal in formulation of anti-obesity treatments (Oertel et al. 2024). PYY mechanics however, were not the same in anorexia nervosa individuals as during an episode of self-restraint from food, plasma PYY levels were elevated (Haines 2023), indicating that the restriction from food was linked specifically to a lack of acknowledgement of bodily needs and absence of self-awareness (Casper 2022). PYY interactions are shown on Fig. 5.

**Box 6: **Fig. 5

### Fasting: Leptin signals in long-term satiety from adipose tissue

**Fig. 6:**
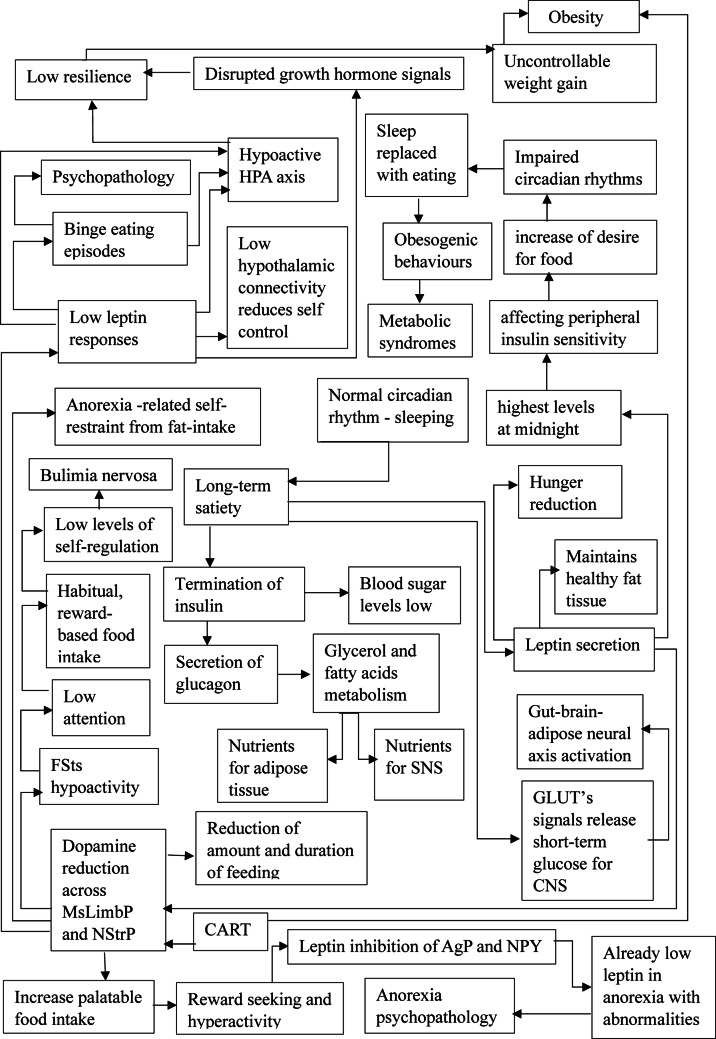
Fasting: GLUT: glucose transporters, MsLimbP: mesolimbic pathway, NStrP – nigrostriatal pathway, FSts: frontostriatal cortex, AgP: agouti-related protein, NPY: Neuropeptide Y; HPA – hypothalamic–pituitary–adrenal axis, CNS: Central nervous system; SNS: sympathetic nervous system

During the long-term satiety, blood sugar levels drop due the termination of insulin secretion and the start of secretion of glucagon. This process stimulates hepatocytes to initiate the chain of glycogenolysis, enabling glycerol and fatty acids to be metabolised within the SNS and deposit the remainder of nutrients into the adipose tissue (Martini et al. 2012). The liver glucose trasporters initiate signals for the short-term preserved glucose from previous meal, to fuel the brain (Blasquez et al. 2014). This phase activates gut-brain-adipose neural axis however, it only operates when the digestive system is empty (Chun-Xia and Tschop 2012). Long-term metabolic phase in eating disorders is of interest because during it is only when the stomach is empty, the adipose tissue cells begin to secrete leptin – a peripheral peptide, known as *the anti-obesity hormone* (Morrison et al. 2005). During long-term satiety, leptin secretion results in hunger reduction and it contributes to the maintenance of a healthy fat tissue while mediating replenishment of bodily energy reservoirs, to the CNS (Bates and Myers 2003). Leptin is a hormone, secreted in pulsatile patterns during this long-term metabolic phase, with its lowest levels in the mid-afternoon hours and highest at midnight with significantly higher release in obese people, during those times (Licinio et al. 1997). Due to this maladaptive pathway, leptin secretion forms a resistance that promotes obesogenic behaviours via affecting peripheral insulin sensitivity therefore increasing the desire for food (Coppari and Bjørbæk 2012). Additionally, if the person maintains the intake of food during night hours, the circadian rhythms are impaired and their replacement with eating behaviours, promotes obesity (Grossjean et al., 2023). Leptin interactions are shown in Fig. 6.

Malfunctioning of leptin receptor results in increased energy storage by exhibiting continuous hyperphagic behaviours, leading to various metabolic syndromes (Bingham et al. 2008). Research recommends restoration of brain-gut disrupted patterns via weight loss endeavours (Strohacker et al. 2014) as well as examination of the quantities and the duration of food intake, feed only when hungry and avoid sleep deprivation in favour of food intake (Soltanieh et al. 2021). Possibly mediated by leptin deficiencies is the dysfunction of the CART system, linked to chronic obesity in some studies (de Macedo et al. 2016). While CART activity impacts the dopamine mesolimbic pathway (see Fig. 4), fasting results in a significant reduction in dopamine expression and less interest in food intake (Kim et al., 2002; Kristensen et al., 1998). However, research shows that CART-firing was significantly increased while presented with highly palatable foods with simultaneous activation of the reward-seeking complex (Hunter et al. 2004), presenting with important implications of the CART complex to hyperactivity and psychopathology (Jean et al. 2012). Another example is with the nature of leptin function, known to inhibit AgP and NPY neuronal connectivity (see Fig 2). It is expected that leptin mechanism of action should exacerbate anorexic signalling (Hasan and Hasan 2011), however, anorexia afflicted people actually are of low leptin levels (Bluher and Mantzoros 2004). This facts confirms abnormal connectivity during food-restrictive behaviours, suggesting anorexia nervosa, psychosomatic origins (Abbate-Daga et al. 2013) (see Fig. 6).

In normal conditions, leptin signalling reduces the rate of dopaminergic neuronal firing in the mesolimbic and striatal areas and across nucleus accumbens, suggesting a decrease in the amount and the duration of feeding (Krugel et al. 2003). High fat intake suppression levels during fasting reduces leptin storage, and increases frequency of binge eating episodes, raising the frequency of food-based reward system responses (Bodell and Keel 2015). Despite increased serum leptin across some conditions such as autoimmune disorders, in bulimia nervosa leptin production is reduced and because leptin levels are sensitive to the reduction of catecholamine reserves (Homan et al. 2014), capacity to self-regulation is obstructed. Apart from the psychiatric complications, this fact is also suggestive of the risk of pathogenesis of autoimmune origin (Raevuori et al., 2014). Further, because leptins require adequate connectivity with leptin-receptor neurons in order to activate hypothalamic structures, the optimal function of HPA axis is lowered (Park and Ahima 2015). As hypothalamic structures normal function depends on the amounts of circulating leptin, such lowered levels of activation further impede self-control and decrease individual's resilience (Aschbacher et al. 2014). Sub-optimal function of leptin pathways dysregulate the signalling of growth hormone, reduce biochemical balance to coping capacity and initiate binge eating episodes, along with an uncontrollable weight gain (Smitka et al. 2013) (see Fig. 6).

In normal conditions, the frontostriatal cortex activity is responsible for control and organisation therefore promoting behavioural flexibility. Frontostriatal metabolism is of research interest due to its prominent activity in bulimia nervosa (Donnely et al. 2018) with functional brain images displaying disorganised firing and marked hypoactivity in the attention-focusing circuits, conveying habitual, reward seeking-behaviours, adversely affecting capacity for self-regulation (Berner and Marsh 2014). These findings form the rationale for bulimia nervosa disordered eating patterns that have been found to persist even after recovery (Steiger et al. 2001). These outcomes could be further linked to risk of autoimmune pathologies with Smitka et al. (2013) studies confirming decreased levels of autoantibodies against both serotonin and dopamine that could potentially explain symptoms of excessive hunger (Smitka et al. 2013). Fig. 6 shows how the regulation of the reward-seeking mechanisms stands out in harnessing the positive autoimmune responses in the pathogenesis of bulimia nervosa (Bodell and Keel 2015; di Bonaventura et al. 2020).

**Box 7: **Fig. 6

## Sympathoadrenal neurofeedback

### Chronic changes in glucose levels and fatty acid oxidation: the mechanisms of glucoprivation and lipoprivation

**Fig. 7:**
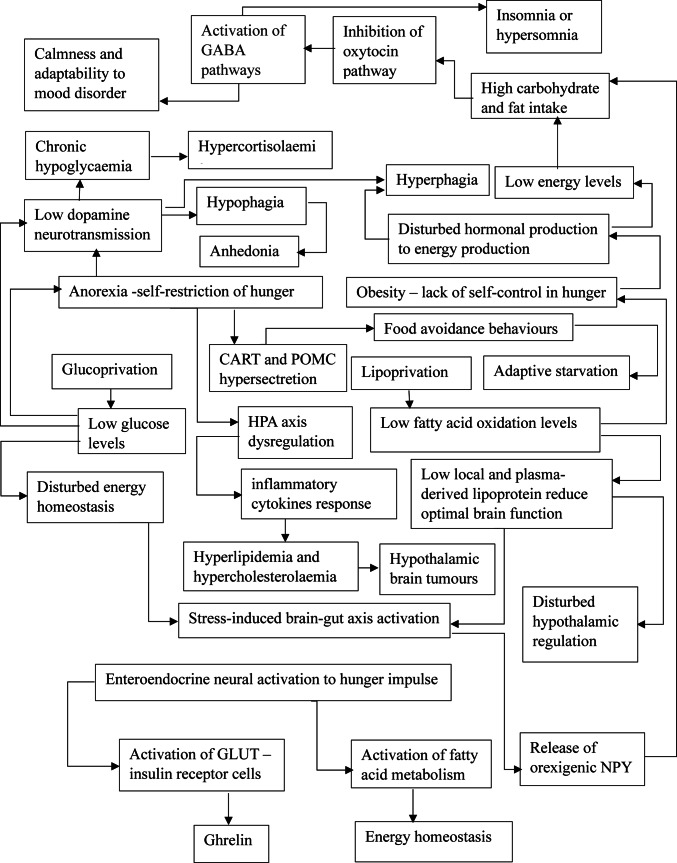
Chronic changes in glucose levels and fatty acids oxidation: the mechanisms of glucoprivation and lipoprivation, CART: cocaine- and amphetamine-regulated transcript; HPA: hypothalamic-pituitary-adrenal axis; GABA: gamma-aminobutyric acid; GLUT: glucose transporters, NPY: neuropeptide Y; POMC: pro-opiomelanocortin

 Glucose and fatty acid oxidation are two neurometabolic processes of the sympathoadrenal system (Jordan et al., 2010), responsible for energy homeostasis. Their signalling is primarily set to increase enteroendocrine production of ghrelin, resulting in potent impulse for hunger and beginning of food intake (Mani et al. 2019). In normal functioning, when the glucose levels reach lower than the required for energy homeostasis, glucoprivation occurs. Its mechanism of action begins with the activation of glucose transporter-insulin receptor cells, signalling hypoglycemic state and triggering impulse for re-feeding (Blasquez et al. 2014). The lipoprivation state is activated when fatty acid oxidation reaches below the level of the organism requirements (Simsek et al. 2014). As the brain is the highest lipid-containing organ after the adipose tissue, the fatty acid metabolism is another prominent neural mechanism in energy homeostasis (Jordan et al. 2010). The lipid content in the brain is derived from both local fatty acid synthesis via lipoprotein receptors as well as from plasma and it influences hypothalamic pathways to regulation of food intake (Kim et al. 2002) (see in Fig. 7). Both glucoprivation and lipoprivation are of interest in studying obesity and anorexia nervosa due to expression of aberrant dopamine neurotransmission (Blum et al. 2014; Sajapitak et al. 2008; Simsek et al., 2014). The mechanism of glucoprivation plays a significant part in the anorexia pathology as it leads to stress-induced hypophagia resulting in chronic hypoglycaemia (Brown and Mehler 2015), that translates into psychological symptom of anhedonia (Qu et al. 2020). Because lipoprivation is related to hypothalamus-regulated energy production and expenditure (Morgan et al. 2004), low energy production further results in chronically increased stress levels, leading to hypercortisolaemia (Lawson et al. 2011) (see Fig. 7).

Physiologically, NPY is significantly involved in the neuromodulation in both glucoprivation and lipoprivation (Kozak et al. 2005), and the alteration of its pathways has been shown to lead to obesity, chiefly from prominent high carbohydrate and fat intake (Li et al. 2020; Rattanajearakul et al. 2025). Neurobiologically, NPY connections activate pathways that inhibit oxytocin release and activate gamma-aminobutyric acid (GABA) pathways which is a connectivity that holds significance to impaired attachment patterns, various somatic symptomatology as well as sleep disturbances as well as implication to psychiatric disorders such as depression (Chaulagain et al. 2025; Huang et al. 2021; Lach et al. 2018; Lawson et al. 2011; Schür et al. 2016). These findings are significant because they show evidence to eating disorders high co-occurence with mood disorders (McAulay et al., 2019), leading to chronic stress adaptation (Herman and Tasker, 2016).

Studies demonstrate a significant relationship between dopamine release and insulin firing on the dopamine-hypothalamus pathway (Stagkourakis et al. 2019). In the brain, glucoprivation is expressed via an enhanced dopamine signalling, associated with intensified metabolic stress (Blum et al. 2014). Since glucose levels are dependent on the optimal function of dopaminergic circuitries, lowered glucose levels inhibit dopamine release, leaving the body dependent on the endogenous dopamine for basal metabolic functions (Adler et al. 2000). As such, chronic states of dopamine depletion in obesity are evidenced by hyperphagic behaviours, in an effort to prevent brain from oxygen starvation (Trugman and James 1993). In anorexia the mechanism of glucoprivation results in hypersecretion of POMC across the CART system and its action lasts longer than in a normal neurometabolic phase (Stoving et al. 1999). Apart from the weight loss, this neural connectivity induces bouts of pleasant mood during appetite-suppressing episodes, resulting in creation of food- avoidance behavioural patterns, and leading to a state of adaptive starvation (Yeomans and Gray 2002).

The interconnectivity with HPA pathway and its degree of activation depends on the levels of lipoprivation (Sajapitak et al. 2008). In anorexia, nervosa, the lipoprivation mechanism malfunctions due to starvation, resulting in increasing serum macrophage inhibitory cytokine-1, inducing inflammatory responses (Karczewska-Kupczewska et al. 2012). As most of the energy needed to initiate gluconeogenesis is obtained from fat oxidation, starvation-induced lipoprivation increases plasma glucose levels (Simsek et al. 2014), carrying both metabolic and neural complications. For example, Ohwada et al. (2006) have found that hypoglycemia-induced inflammation had a simultaneous presence of hyperlipidemia, further indicating accelerated cholesterol metabolism. Some research reports that chronic upgrades in cholesterol accumulation has resulted in tumour formations in lateral hypothalamic area in postmortem hypoglycaemic anorexia nervosa patients (Lewin et al. 1972; Mattingly and Bhanji 1995). These outcomes highlight the necessity of restoring gluconeogenesis and fatty acid metabolism in their simplest form of regulated nutrition, as a beneficial starting point in anorexia treatment (Rosen et al. 2017) (see Fig. 7).

**Box 8: **Fig. 7

### Limitations and considerations for future practice

This paper had some methodological limitations. One of the those was the complexity of problem identification. This study was a preliminary effort to explore the connectivities of neuropathophysiological conditions that have explicit mental health implications. In order maintain validity and reliability of the information, the literature search question had to keep very narrow focus, as some other narrative integrative and descriptive works, with similar topic investigation, have done. Limiting the selection papers to such publications was a restriction however, the narrative integrative literature reviews are designed to address the current state of development of a given topic and to provide a synthesis of the published literature on it (Ferrari 2015). In this light, this narrative presentation is content to offer a *practical yet succinct format* for the novice clinicians in practice.

Another limitation we faced was through data analysis. According to Rumrill and Fitgerald (2001), narrative approach provides room for subjective perceptions in the given field. This review’s goal is to encourage development of newer qualitative perspectives across the domains of the topic. Although this aim could be interpreted as a frivolous change across a methodology by some members of scholarly community, such direction does not necessarily indicate a lack of methodological rigour or flaws in the design (Sukhera 2022), particularly because phenomenological angle in approaching obesity and eating disorders work has recently been recognized as beneficial in exploring newer treatment perspectives (Burns et al. 2017; Chimpen-Lopez and Ariazu-Munoz 2021; Knio and Sridhar 2025).

 The emerging field of neuropathophysiology is an area of knowledge that deals with obesity as a class of medical conditions (Kessler et al. 2016). The key understandings are currently carried out in the emergent research field of hypothalamic neuronal interaction with hormones and gut peptides. This knowledge gives vital information with studies on obesogenic behaviours during periods of stress that involve the brain-gut-adipose neural axis (Agustí et al. 2018; Chun-Xia and Tschop 2012; Weltens et al. 2018). On one hand, NPY is observed to moderate stress centrally, with mechanics of adrenocorticotropic hormone secretion causing corticotropin-releasing hormone to rise across the HPA axis (see Fig. 3), while on the other, this mechanism amplifies stress coping responses peripherally via the activities of the adrenal medulla and sympathetic nerves (Hirsch and Zukowska 2012). This translated into the fact that both obesity and eating disorders remain primarily stress-related conditions and because feeding processes involve production of corticotropin-releasing hormone, directly responsible for energy levels in the body, the eating behaviours during stressful conditions are normally inhibited, as part of normal physiological response to stress. However, it should be cautioned that chronically increased stress levels degrade frontal cortex’ rational executive functions and promotes an emotion-led, stress-based feeding mode (Dallman 2010; George et al. 2010).

The food choices during obesity and eating disorders are generally made during acute emotional episodes where capacity to self-control is reduced. Activation of HPA-induced neuronal firing exacerbates the potential of energy dense and nutrient poor food intake (Pannicke et al. 2021). In hyperphagia, both plasma and fat tissue present with elevated NPY, inducing ravenous desire for food, despite the presence of obesity (Kuo et al. 2008; Lemmens et al. 2011). In obese people, depressive moods such as sadness, feelings of boredom and internal tension have been found to increase habit-based choices of fatty and sweet-tasting, as well as processed foods in contrast to the happy states that favoured dry fruit choices (Dallman 2010; Diz Chaves 2011: Keast et al. 2011). Such examples suggest nutritional recommendation for increased intake of fruits and vegetables due to their verified capacity to positively influence mental health (Głąbska et al. 2020).

Another consideration to obesity prevention are the choices of nutrient-specific foods. For example, oleic fatty acids found in nuts and seeds, avocado and others, were associated with lower adiposity measures, standing against obesity-promoting behaviours (Bauer et al. 2009; Schwartz et al. 2008). Further, oleic fatty acids intake increased production of anti-inflammatory cytokines and secretion of glucagon, resulting in lowering glucose production and leading to overall hunger reduction (Hung et al. 2023), thus, inhibiting food intake and decreasing the release of AgP, reducing the chances for fasting-induced hyperphagia (Quiñones et al. 2015). Furhter, clinical studies demonstrate that neurons in the arcuate nucleus of hypothalamus were affected by the type and the concentration levels of fatty acids (Vohra et al. 2022). For example, oleic acid fails to affect AgP while it depolarises and increases the firing rate of POMC (Michael and Watt, 2020), with both connectivities beneficial to obesity reduction. As the arcuate nucleus of hypothalamus stimulates food intake via hunger-stimulating AgP and inhibits it via hunger-inhibiting POMC (Neudorfer et al. 2020; Roger et al. 2022) (see Fig. 3), oleic acid intake may be of benefit to the treatment of obesity (Tutunchi et al. 2020).

Elenolic acid found in olives and extra virgin olive oil, were confirmed to stimulate PYY release from L-cells (Suzuki et al. 2012). Because PYY is the main nutrient detector organ that sends short-term satiety signal is the liver, its signalling will be maintained for as long as nutrients from stomach and duodenum are received - as it happens during the short-term satiety. However, elenolic acid simultaneously induces secretion of glucagon-like peptide -1 called *liraglutide* in the arcuate nucleus of hypothalamus, effecting upregulations in signalling of the anorexigenic POMC and CART peptides, mediating weight loss processes (Jelsing et al. 2012; Secher et al. 2014). Further, elenolic acid acts as an appetite suppressant towards reduction of food intake volume as well as is of a restorative effect on the stomach tissue, carrying strong implications to maintenance of selected food choices in obesity treatment (Wang et al. 2022).

During trauma recovery, a high level of fatty acids is essential to accelerate the process of generating ketone bodies as an alternative energy source, generally obtained from an increased consumption of fatty acids and decreased carbohydrate intake (Frank and Scolnick 2024). Ketosis has been shown to exert an anorexigenic effect as it increases cholecystokinin signalling and reduces orexigenic signalling such as suppression of hormone ghrelin (Paoli et al. 2015) (see Figs. 4 and 5). Some in vitro studies of brain injuries show such accelerated production of ketones (Cahill Jr. 2006; Dilliraj et al. 2022) with explicit clinically significant results of a reduced onal apoptosis, increase in cognitive capacity, and upregulation across sensorimotor functions (Simeone et al. 2017; Stubbs et al. 2020). As the levels of ketone bodies stands at its highest following bodily starvation during severe traumatic experiences (Daines 2021), the ketogenic diet, combined with low carbohydrate consumption may be recommended for both obesity and bulimia nervosa (Sethi et al. 2020).

Anorexia nervosa’ neuro-endocrine pathologies are an area of continued research interest (Brown and Mehler, 2015; Casper, 2022; Cuntz et al., 2013; Fatt et al., 2020). While hypothalamus influences anorexia' metabolic phases by modifying the humoral and neuronal signals towards energy homeostasis, the modification remains dependent on the level of affect, becasue normally, all emotional, environmental, psychological and physical stresses influence the activity of brain-gut-adipose axis (Yi and Tschop 2012). For example, during an significant emotional stress, traumatic affect or a physically demanding situation, the limbic system initiates autonomic nervous system responses of fight or flight. During these stages, the triglycerides provide up to eighty percent of the bodily energy necessary for the recovery from trauma (Simsek et al. 2014) as evident by a decreased sympathetic activity (Schumann et al. 2017) and the increased rate of lipolysis, both triggered by activation of cortisol and insulin secretions (Polito et al., 2021). Because anorexia nervosa’ psychotic symptomatology is amongst the primary concerns involving increased levels of anxiety along with perpetual alertness (Smitka et al. 2013), evidence-informed psychotherapy interventions (Bower and Irwin, 2016) combined with anxiolytic drug interventions are the prioritised treatment options (Lock and Fitzpatrick 2009).

Because bulimia nervosa significantly affects mental health, the use of standardised clinical questionnaires creates difficulties in some psychotic patients due to the cognitive and emotional aspects of the interviewing protocols (de Beaurepaire 2021). Further, patients with comorbid schizophrenia may misinterpret the purpose of the questions (Palmese et al. 2013) while patients with vagus nerve pathophysiologies could end up feeling depressed, confused or emotionally demanded upon (Faris et al. 2006). Both anorexia nervosa and bulimia nervosa are accepted to largely follow parental autoimmune or autoinflammatory conditions, (Zerwas et al. 2017). Medical literature agrees that eating disorders are complex metabolic, neuroendocrine and psychiatric conditions, with distinct subtypes, involving culture-related differential diagnoses (Hay & Bacaltchuk 2001; Smitka et al. 2013; Zerwas et al. 2017), recommending explorations of heredity, focus on psychoeducation and psychotherapy (Hay 2020).

## Conclusion

Neuroscience is increasingly taking an interest in analytical studies of obesity and eating disorders as conditions of interrelated neural, immune, gastric and mental health factors, with the understanding that brain-gut neurofeedback can inform essential biochemical methods of assessment in support of best treatment choices. A true holistic approach involves consolidation of knowledge within the field of neuropathophysiology while incorporating the essential elements of person-centered counselling, individually tailored nutritional planning and the appopriate consideration of medical management.

## Data Availability

No datasets were generated or analysed during the current study.

## Declarations

### Use of AI

The authors declare there has been no AI used in this review.

## References

[CR1] Abbate-Daga G, Delsedime N, Nicotra B, Giovannone C, Marzola E, Amianto F, Fassino S (2013) Psychosomatic syndromes and anorexia nervosa. BMC Psychiatry 13:14. 10.1186/1471-244X-13-14 23302180 10.1186/1471-244X-13-14 PMC3556145

[CR2] Adler CM, Elman I, Weisenfeld N, Kestler L, Pickar D, Breier A (2000) Effects of acute metabolic stress on striatal dopamine release in healthy volunteers. Neuropsychopharmacology 22(5):545–550. 10.1016/S0893-133X(99)00153-0 10731630 10.1016/S0893-133X(99)00153-0

[CR3] Agustí A, García-Pardo MP, López-Almela I, Campillo I, Maes M, Romaní-Pérez M, Sanz Y (2018) Interplay between the gut-brain axis, obesity and cognitive function. Front Neurosci 12:155. 10.3389/fnins.2018.00155 29615850 10.3389/fnins.2018.00155 PMC5864897

[CR4] Ahima RS, Antwi DA (2008) Brain regulation of appetite and satiety. Endocrinol Metab Clin North Am 37(4):811–823. 10.1016/j.ecl.2008.08.005 19026933 10.1016/j.ecl.2008.08.005 PMC2710609

[CR5] Albracht-Schulte KD, Flynn L, Gary A, Perry CM, Robert-McComb JJ (2023) The physiology of Anorexia Nervosa and Bulimia Nervosa. In: Zumwalt M, Fernandez-del-Valle M, Robert-McComb JJ (eds) The active female. Springer International Publishing, pp 95–117. 10.1007/978-3-031-15485-0_6

[CR6] Alhadeff AL, Mergler BD, Zimmer DJ, Turner CA, Reiner DJ, Schmidt HD, Grill HJ, Hayes MR (2017) Endogenous glucagon-like peptide-1 receptor signaling in the nucleus tractus solitarius is required for food intake control. Neuropsychopharmacology 42(7):1471–1479. 10.1038/npp.2016.246 27782127 10.1038/npp.2016.246 PMC5436110

[CR7] Al-Massadi O, Dieguez C, Schneeberger M, López M, Schwaninger M, Prevot V, Nogueiras R (2021) Multifaceted actions of melanin-concentrating hormone on mammalian energy homeostasis. Nat Rev Endocrinol 17(12):745–755. 10.1038/s41574-021-00559-1 34608277 10.1038/s41574-021-00559-1

[CR8] American Psychiatric Association (APA) (2013) Diagnostic and Statistical Manual of Mental Disorders, 5th Ed. American Psychiatric Publishing, Washington, DC

[CR9] Anderson EJ, Çakir I, Carrington SJ, Cone RD, Ghamari-Langroudi M, Gillyard T, Gimenez LE, Litt MJ (2016) 60 years of POMC: regulation of feeding and energy homeostasis by α-MSH. J Mol Endocrinol 56(4):T157–T174. 10.1530/JME-16-0014 26939593 10.1530/JME-16-0014 PMC5027135

[CR10] Aschbacher K, Rodriguez-Fernandez M, van Wietmarschen H, Tomiyama AJ, Jain S, Epel E, Doyle FJ 3rd, van der Greef J (2014) The hypothalamic-pituitary-adrenal-leptin axis and metabolic health: a systems approach to resilience, robustness and control. Interface Focus 4(5), Article 20140020. 10.1098/rsfs.2014.0020 10.1098/rsfs.2014.0020 PMC4142017 25285198

[CR11] Austin J, Marks D (2009) Hormonal regulators of appetite. Int J Pediatr Endocrinol 2009:141753. 10.1155/2009/141753 19946401 10.1155/2009/141753 PMC2777281

[CR12] Austin A, Flynn M, Richards K, Hodsoll J, Duarte TA, Robinson P, Kelly J, Schmidt U (2021) Duration of untreated eating disorder and relationship to outcomes: a systematic review of the literature. Eur Eat Disord Rev 3:329–345. 10.1002/erv.2745 10.1002/erv.2745 32578311

[CR13] Bailer UF, Kaye WH (2003) A review of neuropeptide and neuroendocrine dysregulation in anorexia and bulimia nervosa. Curr Drug Targets 2(1):53–59. 10.2174/1568007033338689 10.2174/1568007033338689 12769812

[CR14] Barakat GM, Ramadan W, Assi G, Khoury NBE (2024) Satiety: a gut–brain–relationship. J Physiol Sci 74(1), Article 11. 10.1186/s12576-024-00904-9 10.1186/s12576-024-00904-9 PMC10874559 38368346

[CR15] Bates SH, Myers MG Jr (2003) The role of leptin receptor signaling in feeding and neuroendocrine function. Trends Endocrinol Metab 10:447–452. 10.1016/j.tem.2003.10.003 10.1016/j.tem.2003.10.003 14643059

[CR16] Bauer F, Elbers CC, Adan RA, Loos RJ, Onland-Moret NC, Grobbee DE, ... van der Schouw YT (2009) Obesity genes identified in genome-wide association studies are associated with adiposity measures and potentially with nutrient-specific food preference. Am J Clin Nutr 90(4):951–959. https://www.sciencedirect.com/science/article/pii/S0002916523232669 10.3945/ajcn.2009.27781 19692490

[CR17] Berner L, Marsh R (2014) Frontostriatal circuits and the development of bulimia nervosa. Front Behav Neurosci 8(395):1–12. 10.3389/fnbeh.2014.00395 25452718 10.3389/fnbeh.2014.00395 PMC4233924

[CR18] Berthoud HR, Sutton GM, Townsend RL, Patterson LM, Zheng H (2006) Brainstem mechanisms integrating gut-derived satiety signals and descending forebrain information in the control of meal size. Physiol Behav 89(4):517–524. 10.1016/j.physbeh.2006.08.018 16996546 10.1016/j.physbeh.2006.08.018

[CR19] Bingham NC, Anderson KK, Reuter AL, Stallings NR, Parker KL (2008) Selective loss of leptin receptors in the ventromedial hypothalamic nucleus results in increased adiposity and a metabolic syndrome. Endocrinology 149(5):2138–2148. 10.1210/en.2007-1200 18258679 10.1210/en.2007-1200 PMC2329259

[CR20] Blasquez E, Velazquez E, Hurtado-Carneiro V, Ruiz-Albusac JM (2014) Insulin in the brain: its pathophysiological implications for states related with central insulin resistance, Type 2 Diabetes and Alzheimer’s Disease. Front Endocrinol 5(161):1–21. 10.3389/fendo.2014.00161 10.3389/fendo.2014.00161 PMC4191295 25346723

[CR21] Blüher S, Mantzoros CS (2004) The role of leptin in regulating neuroendocrine function in humans. J Nutr 134(9):2469–2474. 10.1093/jn/134.9.2469S 10.1093/jn/134.9.2469S 15333744

[CR22] Blum K, Thanos PK, Gold MS (2014) Dopamine and glucose, obesity, and reward deficiency syndrome. Frontiers in Psychology 5, 919. 10.3389/fpsyg.2014.00919 10.3389/fpsyg.2014.00919 PMC4166230 25278909

[CR23] Bodell LP, Keel PK (2015) <article-title update="modified" original="Weight suppression in bulimia nervosa: Associations with biology and behaviour">Weight suppression in bulimia nervosa: associations with biology and behavior. J Abnorm Psychol 124(4):994–1002. 10.1037/abn0000077 26191637 10.1037/abn0000077 PMC4658277

[CR24] Bower JE, Irwin MR (2016) Mind-body therapies and control of inflammatory biology: a descriptive review. Brain Behav Immun 51:1–11. 10.1016/j.bbi.2015.06.012 26116436 10.1016/j.bbi.2015.06.012 PMC4679419

[CR25] Breit S, Kupferberg A, Rogler G, Hasler G (2018) Vagus nerve as modulator of the brain-gut axis in psychiatric and inflammatory disorders. Front Psychiatry 9:44. 10.3389/fpsyt.2018.00044 29593576 10.3389/fpsyt.2018.00044 PMC5859128

[CR26] Brown C, Mehler PS (2015) Medical complications of anorexia nervosa and their treatments: an update on some critical aspects. Eat Weight Disord 20(4):419–425. 10.1007/s40519-015-0202-3 26138740 10.1007/s40519-015-0202-3

[CR27] Browning KN, Verheijden S, Boeckxstaens GE (2017) The vagus nerve in appetite regulation, mood, and intestinal inflammation. Gastroenterology 152(4):730–744. 10.1053/j.gastro.2016.10.046 27988382 10.1053/j.gastro.2016.10.046 PMC5337130

[CR28] Burns BD, Zhang Y, Wieth M, Touyz S (2017) An exploratory study of creativity and eating disorders. J Eat Disord 5(1), Article 45. 10.1186/s40337-017-0176-9 10.1186/s40337-017-0176-9 PMC5649089 29075495

[CR29] Butterick TA, Billington CJ, Kotz CM, Nixon JP (2013) Orexin: pathways to obesity resistance? Rev Endocr Metab Disord 14(4):357–364. 10.1007/s11154-013-9259-3 24005942 10.1007/s11154-013-9259-3 PMC4739824

[CR30] Casper RC (2022) Restlessness and an increased urge to move (Drive for Activity) in anorexia nervosa may strengthen personal motivation to maintain caloric restriction and may augment body awareness and proprioception: a lesson from leptin administration in anorexia nervosa. Front Psychol 13, Article 885274. 10.3389/fpsyg.2022.885274 10.3389/fpsyg.2022.885274 PMC9359127 35959022

[CR31] Cahill GF Jr (2006) Fuel metabolism in starvation. Annu Rev Nutr 26:1–22. 10.1146/annurev.nutr.26.061505.111258 16848698 10.1146/annurev.nutr.26.061505.111258

[CR32] Chakravorty T (2021) Fat shaming is stopping doctors from helping overweight patients—Here’s what medical students can do about it. BMJ 375:2830–2830. 10.1136/bmj.n2830 10.1136/bmj.n2830 34789468

[CR33] Chaulagain RP, Shrestha Y, Shrestha H, Bhandari R, Gurung P (2025) The neurobiological impact of oxytocin in mental health disorders: a comprehensive review. Ann Med Surg (Lond) 87(3):1479–1486. 10.1097/MS9.0000000000003015 40213210 10.1097/MS9.0000000000003015 PMC11981257

[CR34] Cheng W, Gordian D, Ludwig MQ, Pers TH, Seeley RJ, Myers MG Jr (2022) Hindbrain circuits in the control of eating behaviour and energy balance. Nat Metab 4(7):826–835. 10.1038/s42255-022-00606-9 10.1038/s42255-022-00606-9 35879458

[CR35] Chigbu UE (2019) Visually hypothesising in scientific paper writing: confirming and refuting qualitative research hypotheses using diagrams. Publications (Basel) 7(1):22. 10.3390/publications7010022

[CR36] Chimpén-López C, Arriazu Muñoz R (2021) Narrative therapy for anorexia nervosa: using documents of resistance. Aust N Z J Fam Ther 42(3):276–291. 10.1002/anzf.1459

[CR37] Chun-Xia Y, Tschöp MH (2012) Brain–gut–adipose-tissue communication pathways at a glance. Dis Model Mech 5(5):583–587. 10.1242/dmm.009902 22915019 10.1242/dmm.009902 PMC3424454

[CR38] Cloninger CR (1987) A systematic method for clinical description and classification of personality variants. A proposal. Arch Gen Psychiatry 44(6):573–588. 10.1001/archpsyc.1987.01800180093014 3579504 10.1001/archpsyc.1987.01800180093014

[CR39] Comeras LB, Herzog H, Tasan RO (2019) Neuropeptides at the crossroad of fear and hunger: a special focus on Neuropeptide Y. Ann N Y Acad Sci 1455(1):59–80. 10.1111/nyas.14179 31271235 10.1111/nyas.14179 PMC6899945

[CR40] Conductier G, Nahon JL, Guyon A (2011) Dopamine depresses melanin concentrating hormone neuronal activity through multiple effects on α2-noradrenergic, D1 and D2-like dopaminergic receptors. Neuroscience 178:89–100. 10.1016/j.neuroscience.2011.01.030 21262322 10.1016/j.neuroscience.2011.01.030

[CR41] Concetti C, Peleg-Raibstein D, Burdakov D (2023) Hypothalamic MCH neurons: from feeding to cognitive control. Function 5(1), Article zqad059. 10.1093/function/zqad059 10.1093/function/zqad059 PMC10667013 38020069

[CR42] Coppari R, Bjørbæk C (2012) Leptin revisited: its mechanism of action and potential for treating diabetes. Nat Rev Drug Discov 11(9):692–708. 10.1038/nrd3757 22935803 10.1038/nrd3757 PMC4019022

[CR43] Cuntz U, Enck P, Frühauf E, Lehnert P, Riepl RL, Fichter MM, Otto B (2013) Cholecystokinin revisited: CCK and the hunger trap in anorexia nervosa. PloS ONE 8(1), Article e54457. 10.1371/journal.pone.0054457 10.1371/journal.pone.0054457 PMC3547916 23349895

[CR44] Dahmen N, Becht J, Engel A, Thommes M, Tonn P (2008) Prevalence of eating disorders and eating attacks in narcolepsy. Neuropsychiatr Dis Treat 4(1):257–261. https://pmc.ncbi.nlm.nih.gov/articles/PMC2515918/PMC2515918 18728824

[CR45] Daines SA (2021) The therapeutic potential and limitations of ketones in traumatic brain injury. Front Neurol 12:723148–723148. 10.3389/fneur.2021.723148 34777197 10.3389/fneur.2021.723148 PMC8579274

[CR46] Dallman MF (2010) Stress-induced obesity and the emotional nervous system. Trends Endocrinol Metab 21(3):159–165. 10.1016/j.tem.2009.10.004 19926299 10.1016/j.tem.2009.10.004 PMC2831158

[CR47] de Beaurepaire R (2021) Binge eating disorders in antipsychotic-treated patients with schizophrenia: prevalence, antipsychotic specificities, and changes over time. J Clin Psychopharmacol 41(2):114–120. 10.1097/JCP.0000000000001357 33587392 10.1097/JCP.0000000000001357

[CR48] de Silva A, Bloom SR (2012) Gut hormones and appetite control: a focus on PYY and GLP-1 as therapeutic targets in obesity. Gut Liver 6(1):10–12. 10.5009/gnl.2012.6.1.10 22375166 10.5009/gnl.2012.6.1.10 PMC3286726

[CR49] de Lartigue G, de La Serre CB, Raybould HE (2011) Vagal afferent neurons in high fat diet-induced obesity; intestinal microflora, gut inflammation and cholecystokinin. Physiol Behav 105(1):100–105. 10.1016/j.physbeh.2011.02.040 21376066 10.1016/j.physbeh.2011.02.040 PMC3156356

[CR50] de Macedo IC, de Freitas JS, da Silva Torres IL (2016) The influence of palatable diets in reward system activation: a mini review. Adv Pharmacol Sci 7238679:1–8. 10.1155/2016/7238679 10.1155/2016/7238679 PMC4818794 27087806

[CR51] di Bonaventura MV, Martinelli I, Moruzzi M, Micioni Di Bonaventura E, Giusepponi ME, Polidori C, Lupidi G, Tayebati SK, Amenta F, Cifani C, Tomassoni D (2020) Brain alterations in high fat diet induced obesity: Effects of tart cherry seeds and juice. Nutrients 12(3), Article 1574. 10.3390/nu12030623 10.3390/nu12030623 PMC7146216 32120798

[CR52] Dilliraj LN, Schiuma G, Lara D, Strazzabosco G, Clement J, Giovannini P, Trapella C, Narducci M, Rizzo R (2022) The evolution of ketosis: potential impact on clinical conditions. Nutrients 14(17):3613. 10.3390/nu14173613 36079870 10.3390/nu14173613 PMC9459968

[CR53] Dilsiz P, Aklan I, Sayar Atasoy N, Yavuz Y, Filiz G, Koksalar F, Ates T, Oncul M, Coban I, Ates Oz E, Cebecioglu U, Alp MI, Yilmaz B, Atasoy D (2020) MCH neuron activity is sufficient for reward and reinforces feeding. Neuroendocrinology 110(3–4):258–270. 10.1159/000501234 31154452 10.1159/000501234

[CR54] Diz-Chaves Y (2011) Ghrelin, appetite regulation, and food reward: Interaction with chronic stress. Int J Peptides 898450:1–11. 10.1155/2011/898450 10.1155/2011/898450 PMC3178114 21949667

[CR55] Donnely B, Touyz S, Hay P, Burton A, Russell J, Caterson J (2018) Neuroimaging in bulimia nervosa and binge eating disorder: a systematic review. J Eat Disord 6(3):1–24. 10.1186/s40337-018-0187-1 29468065 10.1186/s40337-018-0187-1 PMC5819247

[CR56] Essner RA, Smith AG, Jamnik AA, Ryba AR, Trutner ZD, Carter ME (2017) AgRP neurons can increase food intake during conditions of appetite suppression and inhibit anorexigenic parabrachial neurons. J Neurosci 37(36):8678–8687. 10.1523/JNEUROSCI.0798-17.2017 28821663 10.1523/JNEUROSCI.0798-17.2017 PMC5588461

[CR57] Faris PL, Eckert ED, Kim SW, Meller WH, Pardo JV, Goodale RL, Hartman BK (2006) Evidence for a vagal pathophysiology for bulimia nervosa and the accompanying depressive symptoms. J Affect Disord 92(1):79–90. 10.1016/j.jad.2005.12.047 16516303 10.1016/j.jad.2005.12.047

[CR58] Fatt SJ, Mond J, Bussey K, Griffiths S, Murray SB, Lonergan A, ... Mitchison D (2020) Help-seeking for body image problems among adolescents with eating disorders: findings from the EveryBODY study. Eat Weight Disorders-Stud Anorexia, Bulimia and Obes 25:1267–1275. https://link.springer.com/article/10.1007/s40519-019-00759-9 10.1007/s40519-019-00759-9 31376110

[CR59] Ferno J, Senaris R, Dieguez C, Tena-Sempere M, Lopez M (2015) Orexins (hypocretins) and energy balance: more than feeding. Mol Cell Endocrinol 418:17–26. 10.1016/j.mce.2015.07.022 26213323 10.1016/j.mce.2015.07.022

[CR60] Ferrari R (2015) Writing narrative style literature reviews. Medical Writing, 24(4):230–235.10.1179/2047480615Z.000000000329

[CR61] Frank GKW, Scolnick B (2024) Therapeutic ketogenic diet as treatment for anorexia nervosa. Front Nutr 11:1392135. 10.3389/fnut.2024.1392135 39296512 10.3389/fnut.2024.1392135 PMC11409850

[CR62] Frank GK, Shott ME, Riederer J, Pryor TL (2016) Altered structural and effective connectivity in anorexia and bulimia nervosa in circuits that regulate energy and reward homeostasis. Transl Psychiatry 6(11):e932. 10.1038/tp.2016.199 27801897 10.1038/tp.2016.199 PMC5314116

[CR63] Fujimoto S, Inui A, Kiyota N, Seki W, Koide K, Takamiya S, Uemoto M, Nakajima Y, Baba S, Kasuga M (1997) Increased cholecystokinin and pancreatic polypeptide responses to a fat-rich meal in patients with restrictive but not bulimic anorexia nervosa. Biol Psychiatry 41(10):1068–1070. 10.1016/S0006-3223(97)00044-9 9129788 10.1016/S0006-3223(97)00044-9

[CR64] George SA, Khan S, Briggs H, Abelson JL (2010) CRH-stimulated cortisol release and food intake in healthy, non-obese adults. Psychoneuroendocrinology 35(4):607–612. 10.1016/j.psyneuen.2009.09.017 19828258 10.1016/j.psyneuen.2009.09.017 PMC2843773

[CR65] Geracioti TD Jr, Liddle RA, Altemus M, Demitrack MA, Gold PW (1992) Regulation of appetite and cholecystokinin secretion in anorexia nervosa. Am J Psychiatry 149(7):958–961. 10.1176/ajp.149.7.958 1609878 10.1176/ajp.149.7.958

[CR66] Głąbska D, Guzek D, Groele B, Gutkowska K (2020) Fruit and vegetable intake and mental health in adults: a systematic review. Nutrients 12(1):115. 10.3390/nu12010115 31906271 10.3390/nu12010115 PMC7019743

[CR67] Greenhalgh T, Thorne S, Malterud K (2018) Time to challenge the spurious hierarchy of systematic over narrative reviews? Eur J Clin Invest 48(6):e12931. 10.1111/eci.12931 29578574 10.1111/eci.12931 PMC6001568

[CR68] Greene JG (2014) Causes and consequences of degeneration of the dorsal motor nucleus of the vagus nerve in Parkinson’s Disease. Antioxidants & Redox Signaling, 21(4):649–667. 10.1089/ars.2014.5859 10.1089/ars.2014.5859 24597973

[CR69] Grill HJ, Hayes MR (2012) Hindbrain neurons as an essential hub in the neuroanatomically distributed control of energy balance. Cell Metab 16(3):296–309. 10.1016/j.cmet.2012.06.015 22902836 10.1016/j.cmet.2012.06.015 PMC4862653

[CR70] Grosjean E, Simonneaux V., Challet E (2023) Reciprocal Interactions between circadian clocks, food intake, and energy metabolism. Biology 12(4):539. 10.3390/biology12040539 10.3390/biology12040539 PMC10136292 37106739

[CR71] Hahn JD (2010) Comparison of melanin-concentrating hormone and hypocretin/orexin peptide expression patterns in a current parceling scheme of the lateral hypothalamic zone. Neurosci Lett 468(1):12–17. 10.1016/j.neulet.2009.10.047 19850103 10.1016/j.neulet.2009.10.047 PMC2800034

[CR72] Haines MS (2023) Endocrine complications of anorexia nervosa. J Eat Disord 11(1):24–24. 10.1186/s40337-023-00744-9 36793059 10.1186/s40337-023-00744-9 PMC9933399

[CR73] Hanssen R, Thanarajah SE, Tittgemeyer M, Brüning JC (2022) Obesity - a matter of motivation? Exp Clin Endocrinol Diabetes 130(5):290–295. 10.1055/a-1749-4852 35181879 10.1055/a-1749-4852 PMC9286865

[CR74] Hara J, Yanagisawa M, Sakurai T (2005) Difference in obesity phenotype between orexin-knockout mice and orexin neuron-deficient mice with same genetic background and environmental conditions. Neurosci Lett 380(3):239–242. 10.1016/j.neulet.2005.01.046 15862893 10.1016/j.neulet.2005.01.046

[CR75] Hasan TF, Hasan H (2011) Anorexia nervosa: a unified neurological perspective. Int J Med Sci 8(8):679–703. 10.7150/ijms.8.679 22135615 10.7150/ijms.8.679 PMC3204438

[CR76] Hay PJ, Bacaltchuk J (2001) Bulimia nervosa. Br Med J 323(7303):33-7. https://www.ncbi.nlm.nih.gov/pmc/articles/PMC3275326/10.1136/bmj.323.7303.33 PMC1120665 11440944

[CR77] Hay P (2020) Current approach to eating disorders: a clinical update. Intern Med J 50(1):24–29. 10.1111/imj.14691 31943622 10.1111/imj.14691 PMC7003934

[CR78] Herman JP, Tasker JG (2016) Paraventricular hypothalamic mechanisms of chronic stress adaptation. Frontiers in endocrinology 7, 137. 10.3389/fendo.2016.00137 10.3389/fendo.2016.00137 PMC5086584 27843437

[CR79] Hirsch D, Zukowska Z (2012) NPY and stress 30 years later: the peripheral view. Cell Mol Neurobiol 32(5):645–659. 10.1007/s10571-011-9793-z 22271177 10.1007/s10571-011-9793-z PMC3492947

[CR80] Holt MK (2022) The ins and outs of the caudal nucleus of the solitary tract: an overview of cellular populations and anatomical connections. J Neuroendocrinol 34(6):e13132. 10.1111/jne.13132 35509189 10.1111/jne.13132 PMC9286632

[CR81] Homan P, Grob S, Milos G, Schnyder U, Eckert A, Lang U, Hasler G (2014) The role of BDNF, leptin, and catecholamines in reward learning in bulimia nervosa. Int J Neuropsychopharmacol 18(5):pyu092. 10.1093/ijnp/pyu092 25522424 10.1093/ijnp/pyu092 PMC4376547

[CR82] Hopia H, Latvala E, Liimatainen, L (2016) Reviewing the methodology of an integrative review. Scand. J Caring Sci 30(4):662–669. 10.1111/scs.12327 10.1111/scs.12327 27074869

[CR83] Hruby A, Hu FB (2015) The epidemiology of obesity: a big picture. Pharmacoeconomics 33(7):673–689. 10.1007/s40273-014-0243-x 25471927 10.1007/s40273-014-0243-x PMC4859313

[CR84] Huang Y, Lin X, Lin S (2021) Neuropeptide Y and metabolism syndrome: An update on perspectives of clinical therapeutic intervention strategies. Front Cell Dev Biol 9:695623. 10.3389/fcell.2021.695623 34307371 10.3389/fcell.2021.695623 PMC8299562

[CR85] Hung H-C, Tsai S-F, Chou H-W, Tsai M-J, Hsu P-L, Kuo Y-M (2023) Dietary fatty acids differentially affect secretion of pro-inflammatory cytokines in human THP-1 monocytes. Sci Rep 13(1):5511–5511. 10.1038/s41598-023-32710-5 37016048 10.1038/s41598-023-32710-5 PMC10073224

[CR86] Hunter RG, Philpot K, Vicentic A, Dominguez G, Hubert GW, Kuhar MJ (2004) CART in feeding and obesity. Trends Endocrinol Metab 15(9):454–459. 10.1016/j.tem.2004.09.010 15519893 10.1016/j.tem.2004.09.010

[CR87] Ilnytska O, Argyropoulos G (2008) The role of the agouti-related protein in energy balance regulation. Cell Mol Life Sci 17:2721–2731. 10.1007/s00018-008-8104-4 10.1007/s00018-008-8104-4 PMC2748318 18470724

[CR88] Jackson PJ, Douglas NR, Chai B, Binkley J, Sidow A, Barsh GS, Millhauser GL (2006) Structural and molecular evolutionary analysis of agouti and agouti-related proteins. Chem Biol 13(12):1297–1305. 10.1016/j.chembiol.2006.10.006 17185225 10.1016/j.chembiol.2006.10.006 PMC2907901

[CR89] Jahan N, Naveed S, Zeshan M, Tahir MA (2016) How to conduct a systematic review: A narrative literature review. Curēus 8(11), Article e864. 10.7759/cureus.864 10.7759/cureus.864 PMC5137994 27924252

[CR90] Jean A, Laurent L, Bockaert J, Charnay Y, Dusticier N, Nieoullon A, Barrot M, Neve R, Compan V (2012) The nucleus accumbens 5-HTR4-CART pathway ties anorexia to hyperactivity. Transl Psychiatry 2(12):e203. 10.1038/tp.2012.131 23233022 10.1038/tp.2012.131 PMC3565192

[CR91] Jelsing J, Vrang N, Hansen G, Raun K, Tang-Christenen M, Knudsen LB (2012) Liraglutide: short-lived effect on gastric emptying - long lasting effects on body weight. Diabetes Obes Metab 14(6):531–538. 10.1111/j.1463-1326.2012.01557.x 22226053 10.1111/j.1463-1326.2012.01557.x

[CR92] Jerlhag E, Egecioglu E, Dickson SL, Andersson M, Svensson L, Engel JA (2006) Ghrelin stimulates locomotor activity and accumbal dopamine-overflow via central cholinergic systems in mice: implications for its involvement in brain reward. Addict Biol 11(1):45–54. 10.1111/j.1369-1600.2006.00002.x 16759336 10.1111/j.1369-1600.2006.00002.x

[CR93] Jordan SD, Könner AC, Brüning JC (2010) Sensing the fuels: glucose and lipid signaling in the CNS controlling energy homeostasis. Cell Mol Life Sci 67(19):3255–3273. 10.1007/s00018-010-0414-7 20549539 10.1007/s00018-010-0414-7 PMC2933848

[CR94] Kanoski SE, Hayes MR, Greenwald HS, Fortin SM, Gianessi CA, Gilbert JR, Grill HJ (2011) Hippocampal leptin signaling reduces food intake and modulates food-related memory processing. Neuropsychopharmacology 36(9):1859–1870. 10.1038/npp.2011.70 21544068 10.1038/npp.2011.70 PMC3154104

[CR95] Karczewska-Kupczewska M, Kowalska I, Nikolajuk A, Adamska A, Otziomek E, Gorska M, Straczkowski M (2012) Hyperinsulinemia acutely increases serum macrophage inhibitory cytokine-1 concentration in Anorexia Nervosa and obesity. Clin Endocrinol Oxf 76(1):46–50. 10.1111/j.1365-2265.2011.04139.x 21645023 10.1111/j.1365-2265.2011.04139.x

[CR96] Kawatani M, Yamada Y, Kawatani M (2018) Glucagon-like peptide-1 (GLP-1) action in the mouse area postrema neurons. Peptides 107:68–74. 10.1016/j.peptides.2018.07.010 30081042 10.1016/j.peptides.2018.07.010

[CR97] Keast DR, O’Neil CE, Jones JM (2011) Dried fruit consumption is associated with improved diet quality and reduced obesity in US adults: National Health and Nutrition Examination Survey, 1999–2004. Nutr Res 31(6):460–467. 10.1016/j.nutres.2011.05.009 21745628 10.1016/j.nutres.2011.05.009

[CR98] Kessler RM, Hutson PH, Herman BK, Potenza MN (2016) The neurobiological basis of binge-eating disorder. Neurosci Biobehav Rev 63:223–238. 10.1016/j.neubiorev.2016.01.013 26850211 10.1016/j.neubiorev.2016.01.013

[CR99] Kim EK, Miller I, Landree LE, Borisy-Rudin FF, Brown P, Tihan T, Townsend CA, Witters LA, Moran TH, Kuhajda FP, Ronnett GV (2002) Expression of FAS within hypothalamic neurons: a model for decreased food intake after C75 treatment. Am J Physiol Endocrinol Metab 283(5):E867–E879. 10.1152/ajpendo.00178.2002 12376313 10.1152/ajpendo.00178.2002

[CR100] Knio L, Sridhar H (2025) Phenomenology of identity: narrative medicine curricula in the care of eating disorders. J Med Humanit. 10.1007/s10912-025-09929-6. (**Advanced Online Publication**) 39869237 10.1007/s10912-025-09929-6

[CR101] Koreshe E, Paxton S, Miskovic-Wheatley J, Bryant E, Le A, Maloney D, Touyz S, Maguire S (2023) Prevention and early intervention in eating disorders: findings from a rapid review. J Eat Disord 11(1):38–38. 10.1186/s40337-023-00758-3 36899428 10.1186/s40337-023-00758-3 PMC9999654

[CR102] Kotz C, Nixon J, Butterick T, Perez-Leighton C, Teske J, Billington C (2012) Brain orexin promotes obesity resistance. Ann N Y Acad Sci 1264(1):72–86. 10.1007/s11154-013-9259-3 22803681 10.1111/j.1749-6632.2012.06585.x PMC3464355

[CR103] Kozak R, Richy S, Beck B (2005) Persistent alterations in neuropeptide Y release in the paraventricular nucleus of rats subjected to dietary manipulation during early life. Eur J Neurosci 21(10):2887–2892. 10.1111/j.1460-9568.2005.04101.x 15926937 10.1111/j.1460-9568.2005.04101.x

[CR104] Krügel U, Schraft T, Kittner H, Kiess W, Illes P (2003) Basal and feeding-evoked dopamine release in the rat nucleus accumbens is depressed by leptin. Eur J Pharmacol 482(1–3):185–187. 10.1016/j.ejphar.2003.09.047 14660021 10.1016/j.ejphar.2003.09.047

[CR105] Kristensen P, Judge ME, Thim L, Ribel U, Christjansen KN, Wulff BS, Clausen JT, Jensen PB, Madsen OD, Vrang N, Larsen PJ, Hastrup S (1998) Hypothalamic CART is a new anorectic peptide regulated by leptin. Nature 393(6680):72–76. 10.1038/29993 10.1038/29993 9590691

[CR106] Kumar R, Rizvi MR, Saraswat S (2022) Obesity and stress: a contingent paralysis. Int J Prev Med 13:95. 10.4103/ijpvm.IJPVM_427_20 35958362 10.4103/ijpvm.IJPVM_427_20 PMC9362746

[CR107] Kuo LE, Czarnecka M, Kitlinska JB, Tilan JU, Kvetnanský R, Zukowska Z (2008) Chronic stress, combined with a high-fat/high-sugar diet, shifts sympathetic signaling toward neuropeptide Y and leads to obesity and the metabolic syndrome. Ann N Y Acad Sci 1148:232–237. 10.1196/annals.1410.035 19120115 10.1196/annals.1410.035 PMC2914537

[CR108] Lach G, Schellekens H, Dinan TG, Cryan JF (2018) Anxiety, depression, and the microbiome: a role for gut peptides. Neurotherapeutics 15(1):36–59. 10.1007/s13311-017-0585-0 29134359 10.1007/s13311-017-0585-0 PMC5794698

[CR109] Larsen JT, Yilmaz Z, Vilhjálmsson BJ, Thornton LM, Benros ME, Musliner KL, Werge T, Hougaard DM, Mortensen PB, Bulik CM, Petersen LV (2021) Anorexia nervosa and inflammatory bowel diseases—Diagnostic and genetic associations. JCPP Advances 1(4):e12036-n/a. 10.1002/jcv2.12036 10.1002/jcv2.12036 PMC10242845 37431410

[CR110] Lau J, Herzog H (2014) CART in the regulation of appetite and energy homeostasis. Front Neurosci 8(313):1–25. 10.3389/fnins.2014.00313 25352770 10.3389/fnins.2014.00313 PMC4195273

[CR111] Lawson EA, Eddy KT, Donoho D, Misra M, Miller KK, Meenaghan E, Lydecker J, Herzog D, Klibanski A (2011) Appetite-regulating hormones cortisol and peptide YY are associated with disordered eating psychopathology, independent of body mass index. Eur J Endocrinol 164(2):253–261. 10.1530/EJE-10-0523 21098684 10.1530/EJE-10-0523 PMC3677777

[CR112] Lemmens SG, Rutters F, Born JM, Westerterp-Plantenga MS (2011) Stress augments food “wanting” and energy intake in visceral overweight subjects in the absence of hunger. Physiol Behav 103(2):157–163. 10.1016/j.physbeh.2011.01.009 21241726 10.1016/j.physbeh.2011.01.009

[CR113] Lewin K, Mattingly D, Millis RR (1972) Anorexia nervosa associated with hypothalamic tumour. BMJ 2(5814):852–857. 10.1136/bmj.2.5814.629 10.1136/bmj.2.5814.629 PMC1788418 5031690

[CR114] Li X, Si H, Chen Y, Li S, Yin N, Wang Z (2020) Effects of fitness qigong and tai chi on middle-aged and elderly patients with type 2 diabetes mellitus. PLoS ONE 15(12):e0243989. 10.1371/journal.pone.0243989 33332396 10.1371/journal.pone.0243989 PMC7746158

[CR115] Licinio J, Mantzoros C, Negrão AB, Cizza G, Wong ML, Bongiorno PB, Chrousos GP, Karp B, Allen C, Flier JS, Gold PW (1997) Human leptin levels are pulsatile and inversely related to pituitary–ardenal function. Nat Med 3(5):575–579. 10.1038/nm0597-575 9142131 10.1038/nm0597-575

[CR116] Lord M N, Subramanian K, Kanoski SE, Noble EE (2021) Melanin-concentrating hormone and food intake control: Sites of action, peptide interactions, and appetition. Peptides, 137, Article 170476. 10.1016/j.peptides.2020.170476 10.1016/j.peptides.2020.170476 PMC8025943 33370567

[CR117] Lock J, Fitzpatrick KK (2009) Advances in psychotherapy for children and adolescents with eating disorders. Am J Psychother 63(4):287–303. 10.1176/appi.psychotherapy.2009.63.4.287 10.1176/appi.psychotherapy.2009.63.4.287 20131739

[CR118] Maniscalco JW, Rinaman L (2018) Vagal interoceptive modulation of motivated behavior. Physiology 33(2):151–167. 10.1152/physiol.00036.2017 10.1152/physiol.00036.2017 PMC5899236 29412062

[CR119] Mani BK, Shankar K, Zigman JM (2019) Ghrelin’s relationship to blood glucose. Endocrinology 160(5):1247–1261. 10.1210/en.2019-00074 30874792 10.1210/en.2019-00074 PMC6482034

[CR120] Marseglia L, Manti S, D’Angelo G, Nicotera A, Parisi E, Di Rosa G, Gitto E, Arrigo T (2014) Oxidative stress in obesity: a critical component in human diseases. Int J Mol Sci 16(1):378–400. 10.3390/ijms16010378 25548896 10.3390/ijms16010378 PMC4307252

[CR121] Martini F, Simmons MJ, Tallitsch RB (2012) Human anatomy, 7th Ed. Pearson, Glenview, IL

[CR122] Mattingly D, Bhanji S (1995) Hypoglycaemia and anorexia nervosa. J Royal Soc Med 88(4), 191–5. https://pubmed.ncbi.nlm.nih.gov/7745563/PMC1295161 7745563

[CR123] McAdams CJ, Smith W (2015) Neural correlates of eating disorders: translational potential. Neurosci Neuroecon 4:35–49. 10.2147/NAN.S76699 26767185 10.2147/NAN.S76699 PMC4707679

[CR124] McAulay C, Hay P, Mond J, Touyz S (2019) Eating disorders, bipolar disorders and other mood disorders: complex and underresearched relationships. J Eat Disord 7:32. 10.1186/s40337-019-0262-2 10.1186/s40337-019-0262-2 PMC6740009 31528342

[CR125] Méquinion M, Langlet F, Zgheib S, Dickson S, Dehouck B, Chauveau C, Viltart O (2013) Ghrelin: central and peripheral implications in anorexia nervosa. Front Endocrinol 4:15. 10.3389/fendo.2013.00015 10.3389/fendo.2013.00015 PMC3581855 23549309

[CR126] Michael NJ, Watt MJ (2020) Long Chain Fatty Acids Differentially Regulate Sub-populations of Arcuate POMC and NPY Neurons. Neuroscience, 451:164–173. 10.1016/j.neuroscience.2020.09.045 10.1016/j.neuroscience.2020.09.045 33002557

[CR127] Mirza M, Das JM (2019) Neuroanatomy, Area postrema. Retrieved from https://www.ncbi.nlm.nih.gov/books/NBK544249/31334969

[CR128] Miller GD (2019) Appetite regulation: Hormones, peptides, and neurotransmitters and their role in obesity. Am J Lifestyle Med 13(6):586–601. 10.1177/1559827617716376 10.1177/1559827617716376 PMC6796227 31662725

[CR129] Mohammadkhani A, Mitchell C, James MH, Borgland SL, Dayas CV (2024) Contribution of hypothalamic orexin (hypocretin) circuits to pathologies of motivation. Br J Pharmacol 181(22):4430–4449. 10.1111/bph.17325 39317446 10.1111/bph.17325 PMC11458361

[CR130] Morgan C, Cone RD (2006) Melanocortin-5 receptor deficiency in mice blocks a novel pathway influencing pheromone-induced aggression. Behav Genet 36(2):291–300. 10.1007/s10519-005-9024-9 16408249 10.1007/s10519-005-9024-9

[CR131] Morgan K, Obici S, Rossetti L (2004) Hypothalamic responses to long-chain fatty acids are nutritionally regulated. J Biol Chem 279(30):31139–31148. 10.1074/jbc.M400458200 15155754 10.1074/jbc.M400458200

[CR132] Morrison CD, Morton GJ, Niswender KD, Gelling RW, Schwartz MW (2005) Leptin inhibits hypothalamic Npy and Agrp gene expression via a mechanism that requires phosphatidylinositol 3-OH-kinase signaling. Am J Physiol Endocrinol Metab 289(6):1051–1057. 10.1016/j.cmet.2005.10.009 10.1152/ajpendo.00094.2005 16046456

[CR133] Mul JD, Yi CX, van den Berg SA, Ruiter M, Toonen PW, van der Elst MC, Voshol PJ, Ellenbroek BA, Kalsbeek A, la Fleur SE, Cuppen E (2010) Pmch expression during early development is critical for normal energy homeostasis. Am J Physiol Endocrinol Metab 298(3):477–488. 10.1152/ajpendo.00154.2009 10.1152/ajpendo.00154.2009 19934402

[CR134] Murphy K, Bloom S (2006) Gut hormones and the regulation of energy homeostasis. Nature 444:854–859. 10.1038/nature05484 17167473 10.1038/nature05484

[CR135] Mustelin L, Silén Y, Raevuori A, Hoek HW, Kaprio J, Keski-Rahkonen A (2016) The DSM-5 diagnostic criteria for anorexia nervosa may change its population prevalence and prognostic value. J Psychiatr Res 77:85–91. 10.1016/j.jpsychires.2016.03.003 27014849 10.1016/j.jpsychires.2016.03.003

[CR136] Myers MG Jr, Heymsfield SB, Haft C, Kahn BB, Laughlin M, Leibel RL, Tschöp MH, Yanovski JA (2012) Challenges and opportunities of defining clinical leptin resistance. Cell Metab 15(2):150–156. 10.1016/j.cmet.2012.01.002 22326217 10.1016/j.cmet.2012.01.002 PMC3281561

[CR137] Nagatani S, Tsukamura H, Murahashi K, Bucholtz DC, Foster DL, Maeda K (1996) Paraventricular norepinephrine release mediates glucoprivic suppression of pulsatile luteinizing hormone secretion. Endocrinology 137(8):3183–3186. 10.1210/endo.137.8.8754737 8754737 10.1210/endo.137.8.8754737

[CR138] National Eating Disorders Collaboration (2023) Eating disorders in Australia*.* https://nedc.com.au/eating-disorders/eating-disorders-explained/eating-disorders-in-australia

[CR139] Neudorfer C, Germann J, Elias GJB, Gramer R, Boutet A, Lozano AM (2020) A high-resolution in vivo magnetic resonance imaging atlas of the human hypothalamic region. Sci Data 7(1):305. 10.1038/s41597-020-00644-6 32934244 10.1038/s41597-020-00644-6 PMC7492465

[CR140] Nevo I, Slonim-Nevo V (2011) The myth of evidence-based practice: towards evidence-informed practice. Br J Soc Work 41(6):1176–1197. 10.1093/bjsw/bcq149

[CR141] Nixon JP, Kotz CM, Novak CM, Billington CJ, Teske JA (2012) Neuropeptides controlling energy balance: orexins and neuromedins. Handb Exp Pharmacol 209:77–109. 10.1007/978-3-642-24716-3_4 10.1007/978-3-642-24716-3_4 PMC4736749 22249811

[CR142] Noble EE, Wang Z, Liu CM, Davis EA, Suarez AN, Stein LM, Tsan L, Terrill SJ, Hsu TM, Jung AH, Raycraft LM, Hahn JD, Darvas M, Cortella AM, Schier LA, Johnson AW, Hayes MR, Holschneider DP, Kanoski SE (2019) Hypothalamus-hippocampus circuitry regulates impulsivity via melanin-concentrating hormone. Nat Commun 10(1):4923. 10.1038/s41467-019-12895-y 31664021 10.1038/s41467-019-12895-y PMC6820566

[CR143] Oertel M, Ziegler CG, Kohlhaas M, Nickel A, Kloock S, Maack C, Sequeira V, Fassnacht M, Dischinger U (2024) GLP-1 and PYY for the treatment of obesity: a pilot study on the use of agonists and antagonists in diet-induced rats. Endocr Connect 13(3):e230398. 10.1530/EC-23-0398 38300808 10.1530/EC-23-0398 PMC10895316

[CR144] Ohwada R, Hotta M, Oikawa S, Takano K (2006) Etiology of hypercholesterolemia in patients with anorexia nervosa. Int J Eat Disord 39(7):598–601. 10.1002/eat.20298 16791856 10.1002/eat.20298

[CR145] Okonkwo O, Zezoff D, Adeyinka A (2020) Biochemistry, Cholecystokinin (CCK)*.* Retrieved from https://www.ncbi.nlm.nih.gov/books/NBK534204/30480943

[CR146] Palmese LB, Ratliff JC, Reutenauer EL, Tonizzo KM, Grilo CM, Tek C (2013) Prevalence of night eating in obese individuals with schizophrenia and schizoaffective disorder. Compr Psychiatry 54(3):276–281. 10.1016/j.comppsych.2012.07.014 22959340 10.1016/j.comppsych.2012.07.014 PMC4088953

[CR147] Pannicke B, Kaiser T, Reichenberger J, Blechert J (2021) Networks of stress, affect and eating behaviour: anticipated stress coping predicts goal-congruent eating in young adults. The Int J Behav Nutr Phys Act 18(1), Article 9. 10.1186/s12966-020-01066-8 10.1186/s12966-020-01066-8 PMC7796605 33422046

[CR148] Paoli A, Bosco G, Camporesi EM, Mangar D (2015) Ketosis, ketogenic diet and food intake control: a complex relationship. Front Psychol 6:27. 10.3389/fpsyg.2015.00027 25698989 10.3389/fpsyg.2015.00027 PMC4313585

[CR149] Park HK, Ahima RS (2015) Physiology of leptin: energy homeostasis, neuroendocrine function and metabolism. Metab, Clin Exp 64(1):24–34. 10.1016/j.metabol.2014.08.004 25199978 10.1016/j.metabol.2014.08.004 PMC4267898

[CR150] Peruzzo B, Pastor FE, Blázquez JL, Schöbitz K, Peláez B, Amat P, Rodríguez EM (2000) A second look at the barriers of the medial basal hypothalamus. Exp Brain Res 132(1):10–26. 10.1007/s002219900289 10836632 10.1007/s002219900289

[CR151] Peters JH, Simasko SM, Ritter RC (2006) Modulation of vagal afferent excitation and reduction of food intake by leptin and cholecystokinin. Physiol Behav 89(4):477–485. 10.1016/j.physbeh.2006.06.017 16872644 10.1016/j.physbeh.2006.06.017

[CR152] Phillipp E, Pirke KM, Kellner MB, Krieg JC (1991) Disturbed cholecystokinin secretion in patients with eating disorders. Life Sci 48(25):2443–2450. 10.1016/0024-3205(91)90379-p 2046469 10.1016/0024-3205(91)90379-p

[CR153] Pissios P, Frank L, Kennedy AR, Porter DR, Marino FE, Liu FF, Pothos EN, Maratos-Flier E (2008) Dysregulation of the mesolimbic dopamine system and reward in MCH-/- mice. Biol Psychiatry 64(3):184–191. 10.1016/j.biopsych.2007.12.011 18281019 10.1016/j.biopsych.2007.12.011

[CR154] Powell-Wiley TM, Poirier P, Burke LE, Després JP, Gordon-Larsen P, Lavie CJ, Lear SA, Ndumele CE, Neeland IJ, Sanders P, St-Onge MP, American Heart Association Council on Lifestyle and Cardiometabolic Health; Council on Cardiovascular and Stroke Nursing; Council on Clinical Cardiology; Council on Epidemiology and Prevention; and Stroke Council (2021) Obesity and cardiovascular disease: a scientific statement from the American Heart Association. Circulation 143(21):984–1010. 10.1161/CIR.0000000000000973 10.1161/CIR.0000000000000973 PMC8493650 33882682

[CR155] Polito R, Messina G, Valenzano A, Scarinci A, Villano I, Monda M, Cibelli G, Porro C, Pisanelli D, Monda V, Messina A (2021) The role of very low calorie ketogenic diet in sympathetic activation through cortisol secretion in male obese population. J Clin Med 10(18):4230. 10.3390/jcm10184230 10.3390/jcm10184230 PMC8470486 34575351

[CR156] Qu N, He Y, Wang C, Xu P, Yang Y, Cai X, Liu H, Yu K, Pei Z, Hyseni I, Sun Z, Fukuda M, Li Y, Tian Q, Xu Y (2020) A POMC-originated circuit regulates stress-induced hypophagia, depression, and anhedonia. Mol Psychiatry 25(5):1006–1021. 10.1038/s41380-019-0506-1 31485012 10.1038/s41380-019-0506-1 PMC7056580

[CR157] Quiñones M, Al-Massadi O, Gallego R, Fernø J, Diéguez C, López M, Nogueiras R (2015) Hypothalamic CaMKKβ mediates glucagon anorectic effect and its diet-induced resistance. Mol Metab 4(12):961–970. 10.1016/j.molmet.2015.09.014 26909312 10.1016/j.molmet.2015.09.014 PMC4731730

[CR158] Raybould HE (2007) Mechanisms of CCK signaling from gut to brain. Curr Opin Pharmacol 7(6):570–574. 10.1016/j.coph.2007.09.006 17954038 10.1016/j.coph.2007.09.006 PMC2692370

[CR159] Rattanajearakul N, Kondoh K, Fu O, Okamoto S, Kobayashi K, Nakajima KI, Minokoshi Y (2025) Glucoprivation-induced nutrient preference relies on distinct NPY neurons that project to the paraventricular nucleus of the hypothalamus. Metab: Clin Exp 156415. Advance online publication. 10.1016/j.metabol.2025.156415 10.1016/j.metabol.2025.156415 41077338

[CR160] Raevuori A, Haukka J, Vaarala O, Suvisaari JM, Gissler M, Grainger M, Linna MS, Suokas JT (2014) The increased risk for autoimmune diseases in patients with eating disorders. PloS one, 9(8): Article e104845. 10.1371/journal.pone.0104845 10.1371/journal.pone.0104845 PMC4141740 25147950

[CR161] Roberts BL, Zhu M, Zhao H, Dillon C, Appleyard SM (2017) High glucose increases action potential firing of catecholamine neurons in the nucleus of the solitary tract by increasing spontaneous glutamate inputs. Am J Physiol 313(3):229–239. 10.1152/ajpregu.00413.2016 10.1152/ajpregu.00413.2016 PMC5625278 28615161

[CR162] Roger C, Lasbleiz A, Guye M, Dutour A, Gaborit B, Ranjeva JP (2022) The role of the human hypothalamus in food intake networks: an MRI perspective. Front Nutr 8:760914. 10.3389/fnut.2021.760914 35047539 10.3389/fnut.2021.760914 PMC8762294

[CR163] Rosen E, Bakshi M, Watters A, Rosen HR, Mehler PS (2017) Hepatic complications of anorexia nervosa. Dig Dis Sci 62(11):2977–2981. 10.1007/s10620-017-4766-9 28932925 10.1007/s10620-017-4766-9

[CR164] Rushing JM, Jones LE, Carney CP (2003) Bulimia nervosa: a primary care review. J Clin Psychiatry 5(5):217–224. 10.4088/pcc.v05n0505 10.4088/pcc.v05n0505 PMC419300 15213788

[CR165] Rumrill J, Fitzgerald SM (2001) Using narrative literature reviews to build a scientific knowledge base. Work 16(2):165–170. 10.3233/WOR-2001-00173 12441470

[CR166] Sajapitak S, Uenoyama Y, Yamada S, Kinoshita M, Iwata K, Bari FY, I’anson H, Tsukamula H, Maeda K (2008) Paraventricular alpha1- and alpha2-adrenergic receptors mediate hindbrain lipoprivation-induced suppression of luteinizing hormone pulses in female rats. J Reprod Dev 54(3):198–202. 10.1262/jrd.20024 18344615 10.1262/jrd.20024

[CR167] Schalla MA, Stengel A (2018) The role of ghrelin in anorexia nervosa. Int J Mol Sci 19(7):2117. 10.3390/ijms19072117 30037011 10.3390/ijms19072117 PMC6073411

[CR168] Schmalbach I, Herhaus B, Pässler S, Runst S, Berth H, Wolff-Stephan S, Petrowski K (2020) Cortisol reactivity in patients with anorexia nervosa after stress induction. Transl Psychiatry 10(1):275. 10.1038/s41398-020-00955-7 32778654 10.1038/s41398-020-00955-7 PMC7417562

[CR169] Schorr M, Miller KK (2017) The endocrine manifestations of anorexia nervosa: mechanisms and management. Nat Rev Endocrinol 13(3):174–186. 10.1038/nrendo.2016.175 27811940 10.1038/nrendo.2016.175 PMC5998335

[CR170] Schumann U, Jenkinson CP, Alt A, Zügel M, Steinacker JM, Flechtner-Mors M (2017) Sympathetic nervous system activity and anti-lipolytic response to iv-glucose load in subcutaneous adipose tissue of obese and obese type 2 diabetic subjects. PLoS ONE 12(3):e0173803. 10.1371/journal.pone.0173803 28346464 10.1371/journal.pone.0173803 PMC5367786

[CR171] Schür RR, Draisma LW, Wijnen JP, Boks MP, Koevoets MG, Joëls M, Klomp DW, Kahn RS, Vinkers CH (2016) Brain GABA levels across psychiatric disorders: a systematic literature review and meta-analysis of (1) H-MRS studies. Hum Brain Mapp 37(9):3337–3352. 10.1002/hbm.23244 27145016 10.1002/hbm.23244 PMC6867515

[CR172] Schwartz GJ, Fu J, Astarita G, Li X, Gaetani S, Campolongo P, Cuomo V, Piomelli D (2008) The lipid messenger OEA links dietary fat intake to satiety. Cell Metab 8(4):281–288. 10.1016/j.cmet.2008.08.005 18840358 10.1016/j.cmet.2008.08.005 PMC2572640

[CR173] Schwartz MW, Seeley RJ, Zeltser LM, Drewnowski A, Ravussin E, Redman LM, Leibel RL (2017) Obesity pathogenesis: an Endocrine Society scientific statement. Endocr Rev 38(4):267–296. 10.1210/er.2017-00111 28898979 10.1210/er.2017-00111 PMC5546881

[CR174] Secher A, Jelsing J, Baquero AF, Hecksher-Sørensen J, Cowley MA, Dalbøge LS, Hansen G, Grove KL, Pyke C, Raun K, Schäffer L, Tang-Christensen M, Verma S, Witgen BM, Vrang N, Bjerre Knudsen L (2014) The arcuate nucleus mediates GLP-1 receptor agonist liraglutide-dependent weight loss. J Clin Invest 124(10):4473–4488. 10.1172/JCI75276 25202980 10.1172/JCI75276 PMC4215190

[CR175] Sethi S, Sinha A, Gearhardt AN (2020) Low carbohydrate ketogenic therapy as a metabolic treatment for binge eating and ultraprocessed food addiction. Curr Opin Endocrinol Diabetes Obes 27(5):275–282. 10.1097/MED.0000000000000571 32773576 10.1097/MED.0000000000000571

[CR176] Simeone TA, Simeone KA, Rho JM (2017) Ketone bodies as anti-seizure agents. Neurochem Res 42(7):2011–2018. 10.1007/s11064-017-2253-5 28397070 10.1007/s11064-017-2253-5 PMC5505793

[CR177] Şimşek T, Şimşek HU, Cantürk NZ (2014) Response to trauma and metabolic changes: posttraumatic metabolism. Turk J Surg 30(3):153–159. 10.5152/UCD.2014.2653 10.5152/UCD.2014.2653 PMC4379844 25931917

[CR178] Smitka K, Papezova H, Vondra K, Hill M, Hainer V, Nevidkova J (2013) The Role of “mixed” orexigenic and anorexigenic signals and autoantibodies reacting with appetite-regulating neuropeptides and peptides of the adipose tissue-gut-brain axis: relevance to food intake and nutritional status in patients with anorexia nervosa and bulimia nervosa. Int J Endocrinol 483145:1–22. 10.1155/2013/483145 10.1155/2013/483145 PMC3782835 24106499

[CR179] Södersten P, Nergårdh R, Bergh C, Zandian M, Scheurink A (2008) Behavioral neuroendocrinology and treatment of anorexia nervosa. Front Neuroendocrinol 29(4):445–462. 10.1016/j.yfrne.2008.06.001 18602416 10.1016/j.yfrne.2008.06.001

[CR180] Soltanieh S, Solgi S, Ansari M, Santos HO, Abbasi B (2021) Effect of sleep duration on dietary intake, desire to eat, measures of food intake and metabolic hormones: a systematic review of clinical trials. Clin Nutr ESPEN 45:55–65. 10.1016/j.clnesp.2021.07.029 34620371 10.1016/j.clnesp.2021.07.029

[CR181] South EH, Ritter RC (1983) Overconsumption of preferred foods following capsaicin pretreatment of the area postrema and adjacent nucleus of the solitary tract. Brain Res 288(1–2):243–251. 10.1016/0006-8993(83)90100-2 6661619 10.1016/0006-8993(83)90100-2

[CR182] Sukhera J (2022) Narrative reviews: Flexible, rigorous, and practical. J Grad Med Educ 14(4):414–417. 10.4300/jgme-d-22-00480.1 10.4300/JGME-D-22-00480.1 PMC9380636 35991099

[CR183] Stagkourakis S, Dunevall J, Taleat Z, Ewing AG, Broberger C (2019) Dopamine release dynamics in the tuberoinfundibular dopamine system. J Neurosci 39(21):4009–4022. 10.1523/JNEUROSCI.2339-18.2019 30782976 10.1523/JNEUROSCI.2339-18.2019 PMC6529860

[CR184] Steiger H, Young SN, Kin NM, Koerner N, Israel M, Lageix P, Paris J (2001) Implications of impulsive and affective symptoms for serotonin function in bulimia nervosa. Psychol Med 31(1):85–95. 10.1017/s003329179900313x 11200963 10.1017/s003329179900313x

[CR185] Steinglass JE, Sysko R, Glasofer D, Albano AM, Simpson HB, Walsh BT (2011) Rationale for the application of exposure and response prevention to the treatment of anorexia nervosa. Int J Eat Disord 44(2):134–141. 10.1002/eat.20784 20127936 10.1002/eat.20784 PMC3638259

[CR186] Støving RK, Hangaard J, Hansen-Nord M, Hagen C (1999) A review of endocrine changes in anorexia nervosa. J Psychiatr Res 33(2):139–152. 10.1016/S0022-3956(98)00049-1 10221746 10.1016/s0022-3956(98)00049-1

[CR187] Strohacker K, McCaffery JM, MacLean PS, Wing RR (2014) Adaptations of leptin, ghrelin or insulin during weight loss as predictors of weight regain: a review of current literature. Int J Obes (2005) 38(3):388–396. 10.1038/ijo.2013.118 10.1038/ijo.2013.118 PMC5357888 23801147

[CR188] Stubbs BJ, Koutnik AP, Goldberg EL, Upadhyay V, Turnbaugh PJ, Verdin E, Newman JC (2020) Investigating ketone bodies as immunometabolic countermeasures against respiratory viral infections. Med (New York, ny) 1(1):43–65. 10.1016/j.medj.2020.06.008 10.1016/j.medj.2020.06.008 PMC7362813 32838361

[CR189] Suzuki K, Jayasena CN, Bloom SR (2012) Obesity and appetite control. Exp Diabetes Res 2012:824305. 10.1155/2012/824305 22899902 10.1155/2012/824305 PMC3415214

[CR190] Tack J, Verbeure W, Mori H, Schol J, Van den Houte K, Huang IH, Balsiger L, Broeders B, Colomier E, Scarpellini E, Carbone F (2021) The gastrointestinal tract in hunger and satiety signalling. United Eur Gastroenterol J 9(6):727–734. 10.1002/ueg2.12097 10.1002/ueg2.12097 PMC8280794 34153172

[CR191] Tanaka M, Yamada S, Watanabe Y (2021) The role of neuropeptide Y in the nucleus accumbens. Int J Mol Sci 22(14):7287. 10.3390/ijms22147287 34298907 10.3390/ijms22147287 PMC8307209

[CR192] Tang TN, Toner BB, Stuckless N, Dion KL, Kaplan AS, Ali A (1998) Features of eating disorders in patients with irritable bowel syndrome. J Psychosom Res 45(2):171–178. 10.1016/s0022-3999(97)00300-0 9753389 10.1016/s0022-3999(97)00300-0

[CR193] Tao YX (2010) The melanocortin-4 receptor: physiology, pharmacology, and pathophysiology. Endocr Rev 31(4):506–543. 10.1210/er.2009-0037 20190196 10.1210/er.2009-0037 PMC3365848

[CR194] Thau L, Reddy V, Singh P (2022) Anatomy, central nervous system. In: StatPearls. StatPearls Publishing. https://pubmed.ncbi.nlm.nih.gov/31194336/31194336

[CR195] Thim L, Nielsen PF, Judge ME, Andersen AS, Diers I, Egel - Mitani M, Hastrup S (1998) Purification and characterisation of a new hypothalamic satiety peptide, cocaine and amphetamine regulated transcript (CART), produced in yeast. FEBS Lett 428(3):263–268. 10.1016/S0014-5793(98)00543-2 9654146 10.1016/s0014-5793(98)00543-2

[CR196] Tian DR, Li XD, Shi YS, Wan Y, Wang XM, Chang JK, Yang J, Han JS (2004) Changes of hypothalamic alpha-MSH and CART peptide expression in diet-induced obese rats. Peptides 25(12):2147–2153. 10.1016/j.peptides.2004.08.009 15572204 10.1016/j.peptides.2004.08.009

[CR197] Tomiyama AJ (2019) Stress and obesity. Annu Rev Psychol 70:703–718. 10.1146/annurev-psych-010418-102936 29927688 10.1146/annurev-psych-010418-102936

[CR198] Turner M (2024) Neurobiological and psychological factors to depression. Int J Psychiatry Clin Pract 28(2):114–127. 10.1080/13651501.2024.2382091 10.1080/13651501.2024.2382091 39101692

[CR199] Tutunchi H, Ostadrahimi A, Saghafi-Asl M (2020) The effects of diets enriched in monounsaturated oleic acid on the management and prevention of obesity: a Systematic review of human intervention studies. Advances in Nutrition 11(4):864–877. 10.1093/advances/nmaa013 10.1093/advances/nmaa013 PMC7360458 32135008

[CR200] Trapp S, Brierley DI (2022) Brain GLP-1 and the regulation of food intake: GLP-1 action in the brain and its implications for GLP-1 receptor agonists in obesity treatment. Br J Pharmacol 179(4):557–570. 10.1111/bph.15638 34323288 10.1111/bph.15638 PMC8820179

[CR201] Trugman JM, James CL (1993) D1 dopamine agonist and antagonist effects on regional cerebral glucose utilization in rats with intact dopaminergic innervation. Brain Res 607(1–2):270–274. 10.1016/0006-8993(93)91516-u 8481802 10.1016/0006-8993(93)91516-u

[CR202] Tyszkiewicz-Nwafor M, Jowik K, Dutkiewicz A, Krasinska A, Pytlinska N, Dmitrzak-Weglarz M, Suminska M, Pruciak A, Skowronska B, Slopien A (2021) Neuropeptide Y and peptide YY in association with depressive symptoms and eating behaviours in adolescents across the weight spectrum: From anorexia nervosa to obesity. Nutrients 13(2):598. 10.3390/nu13020598 33670342 10.3390/nu13020598 PMC7917982

[CR203] Ulrich-Lai YM, Herman JP (2009) Neural regulation of endocrine and autonomic stress responses. Nat Rev Neurosci 10(6):397–409. 10.1038/nrn2647 19469025 10.1038/nrn2647 PMC4240627

[CR204] van der Valk ES, Savas M, van Rossum EFC (2018) Stress and obesity: Are there more susceptible individuals? Curr Obes Rep 7(2):193–203. 10.1007/s13679-018-0306-y 10.1007/s13679-018-0306-y PMC5958156 29663153

[CR205] Vincent RP, le Roux CW (2008) The satiety hormone peptide YY as a regulator of appetite. J Clin Pathol 61(5):548–552. 10.1136/jcp.2007.048488 18441153 10.1136/jcp.2007.048488

[CR206] Vohra MS, Benchoula K, Serpell CJ, Hwa WE (2022) AgRP/NPY and POMC neurons in the arcuate nucleus and their potential role in treatment of obesity. Eur J Pharmacol 915:174611-. 10.1016/j.ejphar.2021.174611 34798121 10.1016/j.ejphar.2021.174611

[CR207] Wang HT, Lu QC, Wang Q, Wang RC, Zhang Y, Chen HL, Zhao H, Qian HZ (2008) Role of the duodenum in regulation of plasma ghrelin levels and body mass index after subtotal gastrectomy. World J Gastroenterol 14(15):2425–2429. 10.3748/wjg.14.2425 18416474 10.3748/wjg.14.2425 PMC2705102

[CR208] Wang Y, Wu Y, Wang A, Wang A, Alkhalidy H, Helm R, Zhang S, Ma H, Zhang Y, Gilbert E, Xu B, Liu D (2022) An olive-derived elenolic acid stimulates hormone release from L-cells and exerts potent beneficial metabolic effects in obese diabetic mice. Front Nutr 9:1051452. 10.3389/fnut.2022.1051452 36386896 10.3389/fnut.2022.1051452 PMC9664001

[CR209] Weltens N, Iven J, Van Oudenhove L, Kano M (2018) The gut-brain axis in health neuroscience: implications for functional gastrointestinal disorders and appetite regulation. Ann N Y Acad Sci 1428(1):129–150. 10.1111/nyas.13969 30255954 10.1111/nyas.13969

[CR210] Whittemore R, Knafl K (2005) The integrative review: Updated methodology. J Adv Nurs 52:546–553. 10.1111/j.1365-2648.2005.03621.x 10.1111/j.1365-2648.2005.03621.x 16268861

[CR211] Yahagi N (2017) Hepatic control of energy metabolism via the autonomic nervous system. J Atheroscler Thromb 24(1):14–18. 10.5551/jat.RV16002 27592630 10.5551/jat.RV16002 PMC5225128

[CR212] Yamada C, Mogami S, Kanno H, Hattori T (2018) Peptide YY causes apathy-like behavior via the dopamine D2 receptor in repeated water-immersed mice. Mol Neurobiol 55(9):7555–7566. 10.1007/s12035-018-0931-1 29429048 10.1007/s12035-018-0931-1 PMC6096978

[CR213] Yeomans MR, Gray RW (2002) Opioid peptides and the control of human ingestive behaviour. Neurosci Biobehav Rev 26(6):713–728. 10.1016/s0149-7634(02)00041-6 12479844 10.1016/s0149-7634(02)00041-6

[CR214] Yi CX, Tschöp MH (2012) Brain-gut-adipose-tissue communication pathways at a glance. Dis Model Mech 5(5):583–587. 10.1242/dmm.009902 22915019 10.1242/dmm.009902 PMC3424454

[CR215] Yoon YR, Baik JH (2015) Melanocortin 4 receptor and dopamine D2 receptor expression in brain areas involved in food intake. Endocrinol Metab 30(4):576–583. 10.3803/EnM.2015.30.4.576 10.3803/EnM.2015.30.4.576 PMC4722414 26790386

[CR216] Zerwas S, Larsen JT, Petersen L, Thornton LM, Quaranta M, Koch SV, Pisetsky D, Mortensen PB, Bulik CM (2017) Eating disorders, autoimmune, and autoinflammatory disease. Pediatrics 140(6):e20162089. 10.1542/peds.2016-2089 29122972 10.1542/peds.2016-2089 PMC5703777

[CR217] Zsombok A, Jiang Y, Gao H, Anwar IJ, Rezai-Zadeh K, Enix CL, Münzberg H, Derbenev AV (2014) Regulation of leptin receptor-expressing neurons in the brainstem by TRPV1. Physiol Rep 2(9):e12160. 10.14814/phy2.12160 25263209 10.14814/phy2.12160 PMC4270226

